# Metagenomic Characterization of Multiple Genetically Modified *Bacillus* Contaminations in Commercial Microbial Fermentation Products

**DOI:** 10.3390/life12121971

**Published:** 2022-11-25

**Authors:** Jolien D’aes, Marie-Alice Fraiture, Bert Bogaerts, Sigrid C. J. De Keersmaecker, Nancy H. C. J. Roosens, Kevin Vanneste

**Affiliations:** Sciensano, Transversal activities in Applied Genomics (TAG), J. Wytsmanstraat 14, 1050 Brussels, Belgium

**Keywords:** genetically modified microorganisms (GMM), *Bacillus*, food enzyme, metagenomic shotgun sequencing, hybrid genome assembly

## Abstract

Genetically modified microorganisms (GMM) are frequently employed for manufacturing microbial fermentation products such as food enzymes or vitamins. Although the fermentation product is required to be pure, GMM contaminations have repeatedly been reported in numerous commercial microbial fermentation produce types, leading to several rapid alerts at the European level. The aim of this study was to investigate the added value of shotgun metagenomic high-throughput sequencing to confirm and extend the results of classical analysis methods for the genomic characterization of unauthorized GMM. By combining short- and long-read metagenomic sequencing, two transgenic constructs were characterized, with insertions of alpha-amylase genes originating from *B. amyloliquefaciens* and *B. licheniformis*, respectively, and a transgenic construct with a protease gene insertion originating from *B. velezensis*, which were all present in all four investigated samples. Additionally, the samples were contaminated with up to three unculturable *Bacillus* strains, carrying genetic modifications that may hamper their ability to sporulate. Moreover, several samples contained viable *Bacillus* strains. Altogether these contaminations constitute a considerable load of antimicrobial resistance genes, that may represent a potential public health risk. In conclusion, our study showcases the added value of metagenomics to investigate the quality and safety of complex commercial microbial fermentation products.

## 1. Introduction

Genetically modified microorganisms (GMM) are frequently employed for manufacturing food and feed microbial fermentation products, such as vitamins, additives, flavors, supplements and enzymes, because of the increase in microbial enzyme production efficiency and/or yield [1]. However, their presence is unauthorized in the final products commercialized in the European Union (EU) food and feed chain (EC/2003/1830). Contaminations with unauthorized GMM may raise serious public health concerns, especially since GMM often carry antimicrobial resistance (AMR) genes, and the ingestion of such contaminated products carries a risk of AMR horizontal gene transfer to pathogens and other gut microbiota.

However, development and implementation of detection methods for unauthorized GMM is problematic, since the dossiers with details concerning their properties and design are confidential, and not available to enforcement laboratories. In previous studies, PCR-based methods, including quantitative PCR (qPCR), were developed to screen samples for the presence of GMM contaminations, based on markers known to be often used in the construction of GMM, such as certain antimicrobial resistance (AMR) genes [2,3,4,5,6] and the shuttle vector pUB110 [7]. Using these methods, up until now three different transgenic constructs with insertions of protease (GMM protease1 and protease2) and alpha-amylase (GMM alpha-amylase1) encoding genes were found in food enzyme (FE) products from different brands, leading to 15 RASFF notifications (https://ec.europa.eu/food/safety/rasff-food-and-feed-safety-alerts/rasff-portal_en (accessed on 12 September 2022)). From some of the FE preparations previously collected on the EU market, *Bacillus velezensis* isolates corresponding to the GMM protease1 could be obtained through microbial isolation experiments, which were subsequently further characterized by whole-genome sequencing (WGS) [8,9]. Apart from this GMM protease1, examples of other unauthorized GMM for which whole genomic characterization was performed remain very limited. To our knowledge, the only other reports of interest within this scope focused on the isolation and characterization of a vitamin B2-producing GM *Bacillus subtilis* strain (RASFF2014.1249) in feed additives [10,11].

In both cases, i.e., the protease1-producing *B. velezensis* [9] and the vitamin B2-producing *B. subtilis* [10], the isolates were initially studied by short-read WGS, resulting in raw reads of 50–600 bp in length. Since one of the main limitations of short reads is that they cannot resolve repetitive regions in the genome, this approach did not allow to completely characterize the nature and location of the genetic modifications. In particular, it could not be unambiguously established whether the transgenic constructs were integrated into the host chromosome, or whether they were present as free plasmids. During follow-up studies [8,11], Illumina short-read and Oxford Nanopore Technologies (ONT) long-read WGS were combined using a hybrid assembly strategy, allowing for complete characterization of both GMM. Hybrid assembly methods leverage the strengths of both sequencing technologies by combining the highly accurate short reads with the long reads that are able to bridge repetitive regions, often resulting in a more complete, reliable, and accurate assembly than can be obtained by only employing either one of the sequencing technologies. In particular, D’aes et al. [8] demonstrated that the GMM protease1 construct in the *B. velezensis* strain is harbored on a high-copy episomal plasmid derived from shuttle vector pUB110 that carries two AMR genes and an insert with a protease encoding gene originating from the *B. velezensis* host strain. The AMR genes, *ant(4′)-Ia* and *bleO*, conferring kanamycin and bleomycin resistance, respectively, were a full-length match to known AMR reference sequences, indicating their completeness and therefore potential functionality. Since the inherent risk of the spreading of AMR genes increases when they are carried on mobile genetic elements such as plasmids, this knowledge is important for the assessment of the potential public health risk associated with a GMM contamination.

These examples showcase the added value of a hybrid assembly approach for isolated GMM strains. However, no isolate carrying either the GM amylase1 or the GM protease2 constructs could be obtained from the FE products, highlighting one of the main bottlenecks of the aforementioned strategies for GMM characterization, namely the required isolation step preceding WGS. Because of the confidentiality of the dossiers describing GMM used to manufacture microbial fermentation products, no prior knowledge is available to enforcement laboratories concerning the required growth conditions to culture the GMM of interest. Even if this information were available, other factors can hamper successful isolation, e.g., microbial competition for growth if several species are present. Alternatively, the GMM may have been genetically altered to render it auxotrophic or impair its ability to persist as viable spores. In some cases, DNA walking allows to investigate transgenic constructs of GMM if no isolates are available, but a minimum of prior information about the DNA walk anchor area is still required, while the size range of the characterized unknown regions close to the DNA walk anchor area is generally limited to a few hundred base pairs [5,6,7]. Shotgun metagenomics enables direct sequencing and analysis of all DNA present in a sample, bypassing the need for isolation and cultivation. Based on a previously characterized vitamin B2-producing GM *B.subtilis* strain, Buytaers et al. [12] delivered a proof-of-concept for the potential of metagenomics using both short- and long-read sequencing for the detection and identification of GMM without performing a prior isolation step. This study also highlighted that this promising approach requires optimization of suitable methods for DNA extraction from a complex matrix, as well as advanced bioinformatics methods for the analysis of the metagenomic data.

The aim of the current study was to investigate the added value of shotgun metagenomic sequencing, using both short-read Illumina sequencing and long-read ONT sequencing, to confirm and extend the analysis results of the classical characterization methods, i.e., qPCR and microbial isolation for complex samples, e.g., contaminated with more than one GMM. Our case study consisted of the complete genomic characterization of four commercial FE products from different brands, three alpha-amylases, and one protease sample. All four samples were contaminated with both GMM protease1, which was isolated and characterized previously [8], as well as with the unculturable GMM alpha-amylase1. Using hybrid assembly, the GMM alpha-amylase1 construct could be completely characterized. Moreover, a previously undetected novel GMM and transgenic construct was identified in the samples, carrying another alpha-amylase encoding gene, which was designated GMM alpha-amylase2. Additionally, three different unculturable *Bacillus* strains were discovered that all carried signs of genetic modifications affecting their sporulation ability, supporting that they are GMM and not incidental natural contaminations. The substantial novel findings of this study highlight the potential of metagenomics for the detection and genomic characterization of both known and novel transgenic constructs and their hosts.

## 2. Materials and Methods

### 2.1. Characterization of Samples via Classical Methods

#### 2.1.1. DNA Extraction from FE Matrix

Four FE products from different brands were selected from previous studies [2,4,6,7,8,13], based on their level of contamination with GMM alpha-amylase1 observed with qPCR (Table 1). Genomic DNA was extracted using the Quick-DNA™ HMW MagBead Kit (ZymoResearch) according to the manufacturer’s instructions. Per extract, 200 mg of the FE product was used. Following a centrifugation of 1 min at 5000× *g*, the supernatant was transferred to a new microcentrifuge tube (mix A) while the pellet was suspended in 100 µL of PBS (Gibco). The latter was centrifuged for 1 min at 5000× *g* and the supernatant was combined with mix A. The pellet was suspended in 1 mL of PBS. After a centrifugation of 1 min at 5000× *g*, the supernatant was discarded and the pellet was suspended in 100 µL of TE buffer 1X (IDTE) and 20 µL of MetaPolyzyme (5 mg/mL; Sigma) for an incubation of 60 min at 37 °C. The digested sample was then added to mix A. After adding 20 μL of 10% SDS (Fisher) and 10 μL of Proteinase K (20 mg/mL), the sample was incubated at 55 °C for 30 min. The sample was then centrifuged for 1 min at 5000× *g*. The supernatant was mixed for 20 min with 800 µL of the Quick-DNA™ MagBinding Buffer and 33 μL of the MagBinding Beads. Following a magnetic bead separation, the supernatant was discarded. The sample was gently mixed for 5 min with 500 μL of the Quick-DNA™ MagBinding Buffer. After a magnetic bead separation, the supernatant was discarded and the sample was mixed with 500 μL of the DNA Pre-Wash Buffer. A magnetic bead separation was applied, the supernatant was discarded and the samples were washed by adding 900 μL of the g-DNA Wash Buffer. Following a magnetic bead separation, the supernatant was discarded and the sample was then air dried for 20 min. Finally, the sample was mixed with 50 μL of the DNA Elution Buffer for 10 min at 55 °C and the eluted DNA was then obtained after a magnetic bead separation step.

Extracted DNA was visualized by capillary electrophoresis using the Tapestation 4200 device with the associated genomic DNA Screen Tape and reagents (Agilent). Each DNA concentration was measured by spectrophotometry using the Nanodrop^®^ 2000 (ThermoFisher, Waltham, MA, USA) and each DNA purity was evaluated using the A260/A280 and A260/A230 ratios. 

#### 2.1.2. Real-Time PCR Assays

DNA from FE products was analyzed using real-time PCR methods specific to a genetically modified (GM) *B. velezensis* producing protease (GMM protease1), a second GMM with a transgenic construct encoding a protease (GMM protease2), and a GMM producing alpha-amylase (GMM alpha-amylase1), developed and published previously [5,9].

Each real-time PCR assay was performed in a standard 25 µL reaction volume containing 1X TaqMan^®^ PCR Mastermix (Diagenode), 400 nM of each primer (Eurogentec), 200 nM of the probe (Eurogentec) and 10 ng of DNA. The real-time PCR program consisted of a single cycle of DNA polymerase activation for 10 min at 95 °C followed by 45 amplification cycles of 15 sec at 95 °C (denaturing step) and 1 min at 60 °C (annealing-extension step). All runs were performed on a CFX96 Touch Real-Time PCR Detection System (BioRad). For each assay, an NTC (no template control) was included.

#### 2.1.3. Bacterial Isolation, DNA Extraction and Isolate WGS

Culturing experiments were performed to characterize potential viable *Bacillus* contaminations in the samples, in addition to the GMM protease1 that was isolated previously [7]. 1 g of the FE product was added to 250 mL of Brain-Heart Infusion broth (Sigma-Aldrich) for an incubation overnight at 30 °C. 100 μL of the culture was plated on nutrient agar (Sigma-Aldrich) without antibiotics for an incubation overnight at 30 °C. 

DNA extracted from isolated bacteria was analyzed by the GMM protease1 qPCR method as described in Section 2.1.2, and the BSG qPCR method specific to the *Bacillus subtilis* group developed previously [13]. DNA from isolates being both positive to the BSG marker and negative to the GMM protease1 marker was extracted as described previously [8,9] to avoid selecting protease GMM1 isolates, which were already extensively characterized [8]. Short-read DNA libraries were prepared using the Nextera XT DNA library preparation kit (Illumina) according to the manufacturer’s instructions. Sequencing was carried out on an Illumina MiSeq system with the V3 chemistry, obtaining 250 bp paired-end reads. The amount of genetic material to load was determined by aiming for a theoretical coverage of 60x per sample, based on the average *Bacillus* genome size of ~4 Mbp.

#### 2.1.4. Isolate Genome Assembly and Characterization

Raw short reads were preprocessed with Trimmomatic 0.38 [14] with the following settings: ILLUMINACLIP:NexteraPE-PE.fa:2:30:10, LEADING:10, TRAILING:10, SLIDINGWINDOW:4:20, MINLEN:50. Quality of raw and preprocessed data was evaluated using FastQC 0.11.5 with default settings. For short-read assembly, Unicycler 0.4.8 [15] was employed, with default settings, and with the following dependencies: SPAdes 3.13.0 [16], Pilon 1.23 [17], Bowtie2 2.3.4.3 [18], samtools 1.9 [19], and blast+ 2.7.1. Assembly statistics were obtained with Quast 5.0.2 [20]. For taxonomic classification, GTDB-Tk 1.5.1 [21] was employed, with --min_perc_aa set to 5, using otherwise default settings, and with FastANI 1.33 [22], FastTree 2.1.11 [23], Mash 2.2 [24], Prodigal 2.6.3 [25], pplacer 1.1.alpha19 [26], and HMMER 3.2.1 as dependencies. Prokka 1.14.5 [27] was used for genome annotation, with default settings. Genotypic AMR detection was performed as described in Bogaerts et al. [28], with one modification, i.e., the National Database of Antibiotic Resistant Organisms (NDARO) (retrieved on 12 Juanuary 2021) was used instead of the ResFinder database.

#### 2.1.5. SNP Typing of Isolates

SNP addresses were extracted with PHEnix 1.4.1 [29] and SnapperDB 1.0.6 [29] with *B. licheniformis* ATCC 9789 and *B. velezensis* Pilsner1-2 as reference genomes for the *B. licheniformis* and *B. velezensis* isolates, respectively, as described by D’aes et al. [8] and Nouws et al. [30]. 

### 2.2. Metagenomic Analysis

#### 2.2.1. DNA Library Preparation and Sequencing

Short-read DNA libraries were prepared using the Nextera XT DNA library preparation kit (Illumina) according to the manufacturer’s instructions. Sequencing was carried out on an Illumina MiSeq system with the V3 chemistry, obtaining 250 bp paired-end reads. The 4 FE sample libraries were analyzed on a MiSeq run together with 3 libraries belonging to another study, amounting to 7 sample libraries in total, in equimolar quantities. Additionally, an entire independent MiSeq run was devoted to sequencing the Coobra sample library to obtain a super-high depth sequencing coverage. 

Long-read DNA libraries were prepared using the ligation sequencing kit (SQK-LSK109; Oxford Nanopore Technologies, Oxford, UK) according to the manufacturer’s instructions. Each FE sample library was loaded on an individual R9 MinION flow cell to be sequenced for 48 h. 

#### 2.2.2. Raw Read Preprocessing and Analysis

Raw short reads were preprocessed with Trimmomatic and quality of raw and preprocessed data was evaluated with FastQC as described in Section 2.1.4. Raw long reads were basecalled with Guppy 5.0.7 in GPU mode, with a super accuracy model, and with q-score based filtering disabled. Filtlong 0.2.0 [31] was applied to raw fastq data to remove reads with an average quality score below 7 and read lengths below 1000 bp. Quality statistics on raw and filtered data were collected with NanoPlot 1.33.0 [32] with default settings.

Exploratory taxonomic classification and visualization of the raw short-read data was performed with Kraken2 2.1.1 [33], and Krona 2.7 [34], respectively. Genotypic AMR detection with KMA [35] on raw short and long reads was performed as described by Bogaerts et al. [28], with one modification, i.e., instead of the ResFinder database, the National Database of Antibiotic Resistant Organisms (NDARO) (retrieved on 12 Juanuary 2021) was used, complemented with an in-house database with a *Bacillus*-specific AMR gene (*catA*, CP023729.1:2725109-2725759), which was not present in NDARO.

#### 2.2.3. Metagenome Assembled Genome (MAG) Assembly and Characterization

Metagenomic hybrid assembly was carried out with OPERA-MS 0.9.0 [36] with the --genome-db argument to provide a custom database, with SPAdes 3.13.0 as short-read assembler, and default settings otherwise. The custom database contained all publicly available nucleotide sequences from the NCBI nucleotide database (August 2021) belonging to the genus *Bacillus* that were circular and/or larger than 3 Mbp, to include a wide range of plasmids and genome assemblies. Apart from SPAdes, the OPERA-MS pipeline had the following dependencies: Samtools 0.1.19, Bwa 0.7.10-r789, Blasr 5.1, Minimap2 2.11-r797 [37], Racon 0.5.0 [38], Mash 2.2, MUMmer 3.23, and Pilon 1.22. 

The clusters produced by OPERA-MS correspond to high-quality conservative metagenome assembled genomes (MAGs) and were used for further analysis. As an alternative approach to obtain MAGs, binning was carried out with MetaBAT2 2.15 [39] with default settings, using as input the metagenomic OPERA-MS assembly, and the short and long reads of the samples, mapped to the metagenomic OPERA-MS assembly. Short reads were mapped end-to-end with Bowtie2 2.3.4.3, with the ‘--sensitive’ preset, while the long reads were mapped with Minimap2 2.17 with the ‘map-ont’ preset. Completeness and contamination rates of both the OPERA-MS and Metabat2 MAGs were estimated with CheckM 1.1.3 [40], with default settings, and with Prodigal 2.6.3 and pplacer 1.1.alpha19 as dependencies. 

For metagenomic long-read only assembly, Canu 2.1.1 [41] was employed, with the following settings: genomeSize = 12,000,000, useGrid = false, corMinCoverage = 0, corOutCoverage = 999, correctedErrorRate = 0.105, corMaxEvidenceCoverageLocal = 10, corMaxEvidenceCoverageGlobal = 10, oeaMemory = 32, redMemory = 32, batMemory = 200, maxThreads = 50, and stopOnLowCoverage = 5. The Canu assemblies were afterwards binned with MetaBAT2, as described above.

Taxonomic classification and annotation of the MAGs was performed with GTDB-Tk as described in Section 2.1.4. Additional ANI values were calculated with FastANI 1.33. 

#### 2.2.4. Whole Genome Alignment-Based Comparisons

Multiple genome alignments were made for the annotated *B. licheniformis* and *B. amyloliquefaciens* MAGs and *B. licheniformis* isolates (see Section 3), with progressiveMauve 20150213 [42] with default settings. The included assemblies were the MAGs (Table 2), and the isolate assemblies in case of *B. licheniformis*, and a number of assemblies from reference strains from the NCBI RefSeq database, based on their similarity to the MAGs according to the output of OPERA-MS, and web-based blastn analysis of selected contigs of the MAGs. The *B. licheniformis* alignment included the following reference strains: ATCC9789 (Accession CP023729), SCDB34 (Accession CP014793), MBGJa67 (Accession CP026522), and YNP1-TSU (Accession CM007615). For the *B. amyloliquefaciens* alignment, the MAGs were complemented with reference strains DSM7 (Accession FN597644, *B. amyloliquefaciens* type strain), HK1 (Accession CP018902), 205 (Accession NZ_CP054415), CC178 (NC_022653), and Y2 (Accession NC_017912). 

#### 2.2.5. Estimation of Depth and Breadth of Coverage of *Bacillus* spp. Chromosomes and Extrachromosomal Elements in the Samples

A pipeline for the calculation of the read depth and breadth of coverage for the *Bacillus* species chromosomes and associated extrachromosomal elements detected in the samples was designed to obtain an estimate of the reads that map uniquely, thereby excluding reads multi-mapping to similar regions in the transgenic constructs or *Bacillus* chromosomes. The reference consisted of *B. licheniformis* ATCC9789, *B. amyloliquefaciens* DSM7, *B. velezensis* 10075, the transgenic constructs of GMM alpha-amylase1 (this study), GMM protease1 (Accession OU015425.1), GMM alpha-amylase2 (this study) and the sequences of plasmid pFL7 (Accession AJ577855), and the putative extrachromosomal linear prophage of the GMM protease1 (Accession OU015426). Short reads of the metagenomic samples were trimmed and filtered with Trimmomatic as described previously, and mapped end-to-end with bowtie2 2.3.4.3, with ‘--sensitive’ presets. Raw long reads were mapped with Minimap2 with the ‘map-ont’ presets. The alignments were filtered with Samtools 1.9 to remove alignments with MAPQ values below 2 or below 60, for the short and long reads, respectively, followed by splitting the alignment file according to the reference with Bamtools 2.5.15. Depth of coverage was calculated with Samtools depth with default settings for each resulting alignment file, after which the mean depth and the breadth of coverage were calculated for each reference with an in-house script. The mean depth of coverage only considered sites with a non-zero depth, i.e., all sites of the reference that were not covered by any uniquely mapping reads were excluded from the calculation. To calculate the breadth of coverage for short reads, only sites with a depth of coverage >2 were taken into account, to avoid counting sites with only or two potentially spuriously mapped reads. For long reads, this cutoff was set to >0 because the reads are longer, and were already filtered very strictly on their MAPQ scores, thus all reads were assumed to map correctly.

#### 2.2.6. Investigation of Long-Read Alignments for Detection of Genomic Deletions in the *B. licheniformis* MAG 

To investigate the presence of putative deletions in the *B. licheniformis* MAG that could support the presence of multiple *B. licheniformis* strains (see Section 3), the long-read alignments were sorted and indexed with Samtools, whereafter they were visualized with Integrative Genomics Viewer 2.4.10 [43]. The alignments were checked manually for the presence of macroscopic deletions. For each sample, the percentage of long reads supporting a certain deletion was calculated by subtracting the estimated coverage at the site of the deletion from the average coverage of the 1000 bp regions surrounding either site of the deletion, followed by dividing this number by the latter coverage.

#### 2.2.7. PCR and Sanger Sequencing to Confirm the Insertion of *cat-amyS* Transgenic Construct in *B. licheniformis*, and Confirm the Presence of sigF and yqfD Deletions in *B. licheniformis* Strains

To confirm some of the metagenomic results, PCR assays targeting the areas of interest, followed by Sanger sequencing, were performed for the samples Coobra and Pureferm. Primers were designed using the software Primer3 [44], resulting in the SigF-F (ATGCAGCCGATTTGAAAGAG) and SigF-R (AAAACTCAGGGCAGGGAAAC) primers for the *sigF* deletion, and in the yqfD-F (CTTCTGCTTTTTCGCCATCTT) and yqfD-R (CCTTTCCTCGTGCAGAAGTC) primers for the *yqfD* deletion (Figures S8 and S9). For the chromosomal insertion of the GMM alpha-amylase2 transgenic construct in *B. licheniformis*, several regions (A–D) were targeted (Figure S10), using (i) the A-F (GCGGGACTATGGATGTTTGT) and A-R (GAGACTGTTGCCTGGACCTC) primers for region A, (ii) the B-F (GGCAGAATACATCCTGCA) and B-R (CAAAGTGTCATCAGCCCTCA) primers for region B, (iii) the C-F (CTGCGGACGTTG*CATA*AATA) and C-R (ATGCAGTGTGTGACGGCTAT) primers for region C, and (iv) the D-F (GGCAGAATACATCCTGCAG) and D-R (TTGATTCCATCCCCCTGTAA) primers for region D.

For each PCR assay, a standard 25 µL reaction volume was applied containing 1X Green DreamTaq PCR Master Mix (ThermoFisher Scientific), 400 nM of each primer (Eurogentec) and 10 ng of DNA. The PCR program consisted of a single cycle of 1 min at 95 °C (initial denaturation) followed by 35 amplification cycles of 30 sec at 95 °C (denaturation), 30 sec at 55 °C (annealing) and 1 min at 72 °C (extension) and finishing by a single cycle of 5 min at 72 °C (final extension). The run was performed on a Swift MaxPro Thermal Cycler (Esco). The PCR products were visualized by electrophoresis on 1% agarose gel (Invitrogen, CA, USA) (100 V, 400 mA, 50 min). The sequencing of the PCR products, purified from agarose gel using the QIAEX II Gel Extraction Kit (QIAGEN), was performed on a Genetic Sequencer 3500 using the Big Dye Terminator Kit v3.1 (Applied Biosystems) according to the manufacturer’s instructions. The generated sequences were analysed using the Clustal Omega software [45] through the web-interface of EBI with default parameters (Figures S6, S7 and S10).

#### 2.2.8. Assembly of Mock Metagenomic Datasets with *B. velezensis* and *B. amyloliquefaciens*

To investigate a putative metagenomic hybrid assembly collapse of *B. velezensis* and *B. amyloliquefaciens* into a single MAG for *B. amyloliquefaciens* or *B. velezensis* (see Section 3), mock Illumina and ONT sequencing datasets were constructed, consisting of publicly available data from a *B. amyloliquefaciens* strain (EA19, Accession Bioproject PRJNA744208), mixed with reads from GMM protease1 isolates [8]. The *B. amyloliquefaciens* Illumina reads were 150 bp in length, as opposed to the 250 bp reads of *B. velezensis*, but this was the best available dataset, since there were no publicly available *B. amyloliquefaciens* datasets for a single strain that comprised both ONT reads as well as Illumina reads of 250 bp. 

The first dataset was composed of *B. amyloliquefaciens* and *B. velezensis* Pilsner1-2 (Accession Biosample SAMEA8478143) reads in a 10/1 ratio to mimic the proportions of the read abundance of both strains as estimated for the Coobra sample. In addition, the datasets were subsampled with seqtk 1.3, prior to mixing them, to approximate the absolute read depth of both strains in the Coobra sample. For *B. amyloliquefaciens*, Illumina and ONT reads were subsampled to 250× and 50×, respectively, based on a genome size of 4.0 Mbp, while for *B. velezensis*, Illumina and ONT reads were subsampled to 25× and 5×, respectively, based on a genome size of 4.35 Mbp.

For the second dataset, read-depth and ratio were chosen in order to approximate the conditions in the Pureferm sample, with 220× and 22× Illumina reads, and 1000× and 100× ONT reads for *B. velezensis* and *B. amyloliquefaciens*, respectively, resulting in a reversed 10/1 ratio compared to the first dataset. To obtain 220× Illumina reads for *B. velezensis*, the datasets were combined for four GMM protease1 isolates that were previously shown to be identical [8], i.e., Pilsner1-1 (Accession Biosample SAMEA8478142), Pilsner1-2 (Accession Biosample SAMEA8478143), Pilsner2-1 (Accession Biosample SAMEA8478144), and Pilsner2-2 (Accession Biosample SAMEA8478145). 

The resulting mock datasets were subjected to metagenomic hybrid assembly with the OPERA-MS pipeline, followed by downstream analysis as described in Section 2.2.3.

## 3. Results 

### 3.1. Characterization of Samples by Classical Methods: qPCR, Microbial Isolation, and WGS

For the four food enzyme (FE) products used in this study, Table 1 lists the results of their characterization with classical methods, including qPCR on the FE matrix, and microbial isolation from the FE matrix, followed by WGS-based analysis. qPCR assays were performed for three previously characterized transgenic constructs with insertions of protease (GMM protease1 and GMM protease2) and alpha-amylase (GMM alpha-amylase1) encoding genes. Based on these results, a cross-contamination of food enzyme products with two different GMM, namely GMM protease1 and GMM alpha-amylase1, was demonstrated. 

Microbial isolation experiments were performed to characterize any viable *Bacillus* strains contaminating the samples (see Supplementary text S1 and Table S1 for analysis metrics and a more detailed description of the results). This yielded isolates for samples Coobra and Pureferm, while from samples Stillspirits and Browin no viable strains could be retrieved under the tested conditions. For the Pureferm sample, all 3 isolates obtained in this study corresponded to the GM *B. velezensis* protease1 host strain. However, no sequence related to the GMM protease1 construct (pUB110 shuttle vector and associated AMR genes) was detected in the assemblies, which could likely be explained by the loss of the plasmid carrying the GMM protease1 construct due to the absence of antibiotic selection pressure during the microbial isolation experiment. For the Coobra samples, all 10 isolates obtained in this study were identified as clones of a single *Bacillus licheniformis* strain. No elements associated with the presence of a transgenic construct were identified in the assemblies of these isolates, indicating that it is either not a GMM or alternatively also might have lost the construct due to the absence of a suitable antibiotic selection pressure during the isolation. 

### 3.2. Characterization of Samples Using Shotgun Metagenomic Sequencing and Hybrid Assemblies

#### 3.2.1. The Metagenomic Approach Confirms the Presence of All GMM Contaminations Observed by qPCR

Metagenomic sequencing was carried out to obtain both short- and long-read data, for which key metrics are listed in Table S2, while Figure S1 shows the taxonomic classification results for the raw short reads. Table 2 shows the main metrics of the hybrid metagenomic assemblies and derived MAGs for the four samples. An overview of the extrachromosomal elements, e.g., plasmids, detected in the hybrid metagenomic assemblies is provided in Table S3.

The presence of contaminations related to known GMM was investigated and found to be in line with the qPCR analysis (Section 4.1). In the metagenomic assemblies of the three alpha-amylase FE products, i.e., Coobra, Stillspirits, and Browin, contigs covering the complete GMM alpha-amylase1 construct were detected (Table S3). Additionally, in the protease FE sample Pureferm, a contig partially covering the GMM alpha-amylase1 construct was present, supporting the qPCR result and confirming that Pureferm is cross-contaminated with GMM alpha-amylase1. Conversely, all alpha-amylase sample assemblies displayed contigs with at least a partial GMM protease1 construct (Table S3), confirming the qPCR result and the cross-contamination of these samples with the protease-producing GMM.

#### 3.2.2. Metagenomic Analysis Allows Full Characterization of the Construct of the Unculturable Previouslyidentified GMM Alpha-Amylase1 and the AMR Genes in the Samples 

##### 3.2.2.1. The GMM Alpha-Amylase1 Construct Carries Intact AMR Genes and Is Likely a High-Copy Plasmid

The GMM alpha-amylase1 construct has previously been partially characterized using DNA walking [7], but as no isolate could be obtained for this GMM, complete sequencing and characterization of the construct, and determination of its location (chromosomal or plasmidic) had remained elusive. 

**Figure 1:**
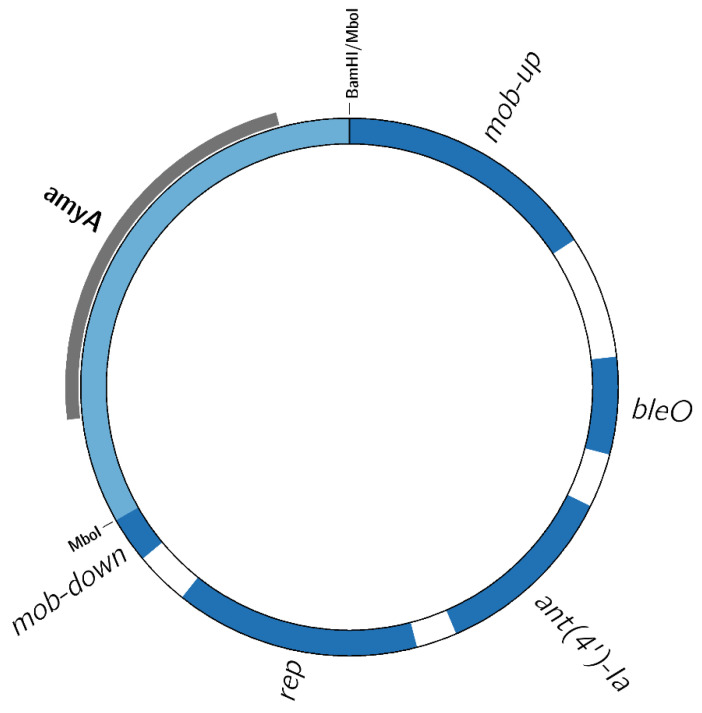
Map of the recombinant 6814 bp GMM alpha-amylase1 construct found in the FE samples. *ant(4′)-Ia*: kanamycin and neomycin resistance gene, *bleO*: bleomycin resistance gene, *rep*: replicase, *mob*: mobilization protein. The *mob* gene is disrupted by a recombinant insert of 2265 bp in length, encompassing the complete alpha-amylase encoding gene *amyA* originating from *B. amyloliquefaciens*, highlighted in grey. BamHI/MboI and MboI indicate the restriction sites that flank the recombinant insert. Figure created with Circos 0.69–6 [47].

With the metagenomic approach, genomic material covering the entire construct and its genomic context could be obtained through metagenomic hybrid assembly. The metagenomic assemblies presented contigs representing at least a partial, in case of the Pureferm sample, or the complete GMM alpha-amylase1 construct, for samples Coobra, Stillspirits, and Browin, allowing for a complete characterization (Figure 1). The complete construct was 6814 bp in length, and derived from shuttle vector pUB110 (Accession M19465, 4548 bp), with a recombinant insert of 2265 bp in length. This insert carried *amyA*, encoding alpha-amylase, and was a nearly 100% identical match to *amyA* of *B. amyloliquefaciens* DSM7 (Accession FN597644). The GMM alpha-amylase1 construct carried two AMR genes: *ant(4′)-Ia*, encoding an aminoglycoside O-nucleotidyltransferase conferring kanamycin and neomycin resistance, and *bleO*, conferring bleomycin resistance. Both AMR genes were a full-length 100% identical match to the reference AMR genes, indicating that they were complete and potentially functional (Tables S4 and S5). The upstream junction of pUB110 and the insert displayed an *Mbo*I restriction site (GATC), while the downstream junction showed a hybrid *Bam*HI/*Mbo*I restriction site (GGATCC) (Figure 1). The recombinant insert disrupted only the *mob* gene, leaving all elements required for normal replication intact [46]. 

Although this could not be unequivocally established, both the available experimental evidence, as well as literature reports, indicated that the GMM alpha-amylase1 is most likely harbored on a free high-copy plasmid (Supplementary text S2). 

##### 3.2.2.2. The Unauthorized GMM Contaminations in the FE Samples Are Associated with a Considerable Load of AMR Genes

AMR gene detection analysis based on the complete metagenomic short-read and long-read datasets, which both show the same trends, as well as on the metagenomic assemblies and MAGs, highlighted that the microbial contamination of the FE samples is associated with a significant presence of AMR genes, both on plasmids as well as of chromosomal origin (Tables S4 and S5). These include the AMR genes associated with the transgenic constructs (Section 3.2.2 and Section 3.2.3.3), but also a number of additional AMR genes, associated with the *Bacillus* host chromosomes and likely of natural origin (Supplementary text S3).

#### 3.2.3. Metagenomic Analysis Reveals the Presence of Novel Unculturable Genetically Modified *Bacillus* strains and of a Novel Transgenic Construct 

In addition to the confirmation of the qPCR results targeting known GM constructs, and the complete characterization of the GMM alpha-amylase1 construct reported in the previous sections, the metagenomic hybrid assembly approach revealed that all samples were contaminated with multiple different *Bacillus* strains, several of which were not previously detected by microbial isolation experiments (Table 2). Moreover, the metagenomic analysis facilitated the discovery and complete characterization of a previously unknown transgenic construct.

Unlike the *B. velezensis* GMM protease1 host strain, the *B. licheniformis* and *B. amyloliquefaciens* strains are unculturable strains that could not be detected with classical (culturing based) analysis methods. In these cases, the culturing conditions may not have been suitable to obtain isolates, or the contaminations may have been solely represented by dead vegetative cells, or even only by free DNA that was released from dead cells. Irrespective of whether viable cells were still present, if the organism could not be cultured, it was designated as ‘unculturable’ for the purpose of this study.

##### 3.2.3.1. Two Unculturable *Bacillus licheniformis* Strains Are Likely Asporogenic GMM 

A single metagenome assembled genome (MAG) for *B. licheniformis* was obtained for all four samples, and in samples Coobra, Browin, and Stillspirits, it was the dominant contamination in terms of read abundance, as indicated by the read-depth reported by OPERA-MS for the different MAGs (Table 2). The *B. licheniformis* OPERA-MS MAGs were 4.05–4.16 Mbp in length, and deemed of high quality, being at least 96% complete. Whole-genome comparison of the *B. licheniformis* MAGs with selected *B. licheniformis* reference genomes (see Section 2) indicated that the unculturable *B. licheniformis* is closely related to *B. licheniformis* ATCC9789 (Accession CP023729). *B. licheniformis* ATCC9789 is a non-auxotrophic, wild-type strain, which is available for purchase from a number of culture collections. The *B. licheniformis* MAGs and the genome of strain ATCC9789 share a number of genomic islands that are absent from the other strains included in the whole-genome comparison (Figure S2), supporting their close relatedness. Additionally, average nucleotide identity (ANI) estimations between the *B. licheniformis* MAGs and strain ATCC9789 were >99.97% in all cases.

Moreover, in-depth analysis based on inspection of long-read alignments (Supplementary text S4) indicated that in samples Coobra, Stillspirits and Browin, the *B. licheniformis* MAG does not represent one, but two closely related strains, only distinguishable by the presence of a different set of genomic deletions (Table S6). Sample Pureferm on the other hand appeared to be contaminated with only one of the unculturable *B. licheniformis* strains. Additionally, evidence was found, which was supported by PCR, that the two unculturable *B. licheniformis* strains were genetically modified to impair their ability to sporulate (Supplementary text S4). More specifically, the *B. licheniformis* strain that was found in all four FE samples carried a deletion affecting sporulation genes *sigF* and *spoIIAB* (Figures S3, S4 and S7). The other strain, detected in the alpha-amylase FE samples Coobra, Browin, and Stillspirits, but not Pureferm, harbored a deletion in the *yqfD* sporulation gene (Figures S5 and S6).

Finally, whole-genome comparison clearly demonstrated that the viable *B. licheniformis* strain that was isolated from the Coobra sample (Section 3.1 and Supplementary text S1) is distinct from the unculturable *B. licheniformis* strains, as illustrated in Figure S2. Furthermore, none of the deletions found in the unculturable *B. licheniformis* strains (Table S6) were detected in the isolate assemblies, underpinning their difference.

##### 3.2.3.2. An Unculturable *Bacillus amyloliquefaciens* Strain Is Potentially an Asporogenic GMM

In the samples Coobra and Stillspirits, two incomplete, distinct OPERA-MS MAGs per sample were classified as *B. amyloliquefaciens*, while MetaBAT2 outputted a single *B. amyloliquefaciens* MAG for Coobra, Stillspirits, as well as for Browin (Table 2), albeit a highly incomplete one. For Pureferm, no *B. amyloliquefaciens* MAG was generated at all, although read-mapping analysis suggested that *B. amyloliquefaciens* is present at a low abundance (Table S7). A potential explanation for these inconsistent results is the occurrence of an assembly collapse of the highly similar genomes of the *B. velezensis* strain (GMM protease 1) with that of the *B. amyloliquefaciens* strain, as supported by assembly of mock metagenomic datasets containing both *B. velezensis* and *B. amyloliquefaciens* reads with uneven relative abundances. Overall, the analysis indicated that only one *B. amyloliquefaciens* strain was present in the samples, despite the output of two separate MAGs by OPERA-MS (Supplementary text S6).

The MetaBAT2 MAGs of Coobra and Stillspirits were included in a whole-genome comparison with a selection of *B. amyloliquefaciens* reference strains (see Section 2). This revealed the presence of a 6 bp insertion in *sigK*, also known as *spoIIIC*, encoding a sigma factor responsible for the expression of sporulation specific genes, in the *B. amyloliquefaciens* MAGs of Coobra and Stillspirits, compared to the reference strains. The insertion is not present in the *B. velezensis* GMM isolate genome, confirming that it is not an assembly artefact resulting from the presence of the two similar strains (see Supplementary text S6). The insertion might therefore represent a genuine and unique genetic modification to impair the sporulation ability of the strain, similar to the unculturable *B. licheniformis* strains described in Section 3.2.3.1. Analysis of the predicted protein sequence of the gene showed that it constitutes an in-frame mutation, resulting in the insertion of ‘NA’ in the primary sequence of the protein. The possibility that this mutation occurred naturally cannot be excluded, although further investigation indicated it was never present in any of the publicly available *B. amyloliquefaciens* genomes in NCBI. Apart from the *sigK* insertion, no other conspicuous putative modifications were found that could indicate this strain potentially being genetically modified.

##### 3.2.3.3. A Novel GMM Alpha-Amylase2 Construct Is Integrated into the Genome of the Unculturable *B. licheniformis*

**Figure 2:**
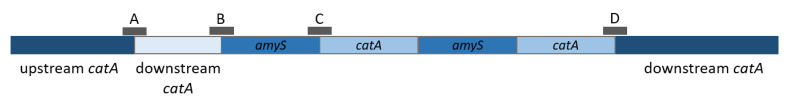
Map of the *GMM alpha-amylase2* construct, of which at least two contiguous copies are integrated into the genome of its *B. licheniformis* host at the site of the wild-type *catA* gene. *amyS*: gene encoding alpha-amylase from *B. licheniformis*, *catA*: gene encoding type A chloramphenicol O-acetyltransferase. The dark grey bars indicate the amplicons of the PCR assays targeting the junctions of the different components of the construct, details for which are provided in Figure S10. A single copy of the construct is 3606 bp in length, and composed of two sequences originating from *B. licheniformis*: a region encompassing the *amyS* gene (nt 652,532-654,717 in reference ATCC 9789), linked to a region encompassing the *catA* gene (nt 2,725,048–2,726,467 in reference ATCC 9789). The particular composition of this region, with a duplication of the sequence immediately downstream of *catA*, is likely the consequence of two contiguous genetic modifications: first the deletion of *catA* from the host chromosome, followed by insertion of the transgenic construct.

Our investigation (Supplementary text S5, Figures S8–S10, Table S8) revealed the presence of an additional transgenic construct in all four samples, which was not previously detected using the classical qPCR- or isolation-based methods. This construct (Figure 2), designated GMM alpha-amylase2, carried the *catA* AMR gene, flanked by an amylase encoding gene (*amyS*) originating from *B. licheniformis*, and not from *B. amyloliquefaciens* as is the case for the GMM alpha-amylase1 construct. The *B. licheniformis amyS* gene shares only 74% nucleotide sequence identity with its alpha-amylase encoding counterpart *amyA* from *B. amyloliquefaciens*.

*catA* is an AMR gene, encoding a type A chloramphenicol O-acetyltransferase that has recently been described in literature as being common in *B. (para)licheniformis* [48], and is phylogenetically distinct to previously described *catA* from other bacterial species. The *catA* gene in the novel construct was a full-length 100% identical match to the reference from *B. licheniformis* ATCC9789 (Tables S4 and S5), indicating that it is complete and potentially functional.

The results proved (Supplementary text S5) that the GMM alpha-amylase2 construct is integrated into the genome of at least one and potentially both unculturable *B. licheniformis* strains (Figure 2). The available evidence (Supplementary text S5) indicates that the copy number of the construct is at least two, and probably more.

### 3.3. High-Depth Metagenomic Sequencing and Hybrid Assembly Highlights the Presence of GMM Protease1 Host Strain in the Coobra Sample

Despite the positive qPCR signal for GMM protease1 in the alpha-amylase FE samples, a *B. velezensis* MAG, representing the GMM protease1 host strain, was not detected in the assemblies of these samples. To assess the added value of very high depth sequencing, an additional entire independent MiSeq run was carried out, dedicating the full capacity to the Coobra sample to obtain super high (short-read) coverage. The data was analyzed with the same approach as for the smaller datasets. For the hybrid assembly, the data was combined with the same long-read dataset for Coobra as described above. In addition to the unculturable *B. licheniformis* and *B. amyloliquefaciens* MAGs that were also assembled with the lower depth data, this assembly (Table 3) additionally showed two MAGs, classified as *B. velezensis*, i.e., the host species of the GMM protease1 construct. However, even at this high depth, the *B. velezensis* MAGs were of low quality. This may be explained by assembly collapse of the closely related *B. amyloliquefaciens* and *B. velezensis* strains in the samples (Supplementary text S6, Table S9). Furthermore, the high-depth Coobra assembly contained a contig displaying the completely assembled extrachromosomal prophage of the GMM protease1, which in a previous study was shown to be a characteristic element of the genome of this GMM [8], while the lower-depth alpha-amylase datasets of Coobra, Stillspirits and Browin only allowed assembly of small fragments of this prophage (Table S3). These findings provided strong support for the presence of the GMM protease1 host strain in the sample. 

**Figure 3:**
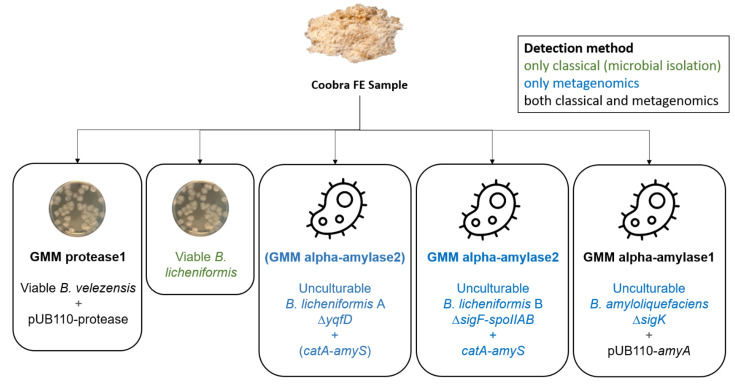
Overview of the contribution of different analysis approaches to the elucidation of the genomic composition of the FE sample Coobra. Metagenomic analysis confirmed the presence of the GMM protease1 and GMM alpha-amylase1 construct and allowed for complete characterization of the latter. Additionally, metagenomics revealed the presence of three unculturable *Bacillus* strains with genetic modifications affecting their sporulation ability, and one novel transgenic construct/GMM (GMM alpha-amylase2). The viable GMM protease1 (*B. velezensis*) strain was characterized previously [8]. The transgenic construct of GMM alpha-amylase1 (pUB110-*amyA*), for which the association with its host strain could not be established with full certainty (see Section 4) is indicated with its most likely host.

Overall, the analysis of the Coobra sample, with a combination of classical analysis methods and in-depth metagenomic analysis, provided a thorough insight into the GMM contaminations in the sample, clearly highlighting the added value and potential of this approach for the investigation of unauthorized GMM contaminations (Figure 3).

## 4. Discussion

In this case, study, the characterization of GMM contaminations in FE products by classical methods, i.e., qPCR and microbial isolation followed by WGS, was compared and complemented with an approach using shotgun metagenomic sequencing with both short- and long-read technologies. Table 4 shows an overview of the most important findings for the Coobra sample, which was studied the most extensively.

The qPCR assays demonstrated the presence of a cross-contamination of the four investigated samples with two previously described known GMM: GMM protease1 and GMM alpha-amylase1. For GMM protease1, viable isolates could be obtained from some of the samples, which were characterized in a previous study using WGS [8]. Microbial isolation experiments were also pivotal to the detection of a viable *B. licheniformis* strain in the Coobra sample, which constitutes a significant unauthorized contamination, even if no signs of genetic modification were observed.

With the metagenomic approach, the presence of GMM contaminations related to the known GMM protease1 and GMM alpha-amylase1 was confirmed, in agreement with the qPCR analysis. Without any prior microbial strain isolation, the transgenic GMM alpha-amylase1 construct could be completely characterized. The genetic make-up of this construct is consistent with that of pKTH10, a recombinant plasmid generated by cloning a *Mbo*I-restriction fragment of approximately 2.3 kb into *Bam*HI-restricted pUB110 [49]. Transformation of a *B. subtilis* host with this plasmid led to a 2500-fold increase of the alpha-amylase activity, according to Palva [49]. To our knowledge, the sequence of pKTH10 was never published, but the close resemblance nevertheless indicates that the design of GMM alpha-amylase1 could potentially be inspired by that of pKTH10. 

While the classical approach with qPCR can detect specific AMR genes for which an assay is available, metagenomics allowed obtaining a complete characterization of the AMR genes in the samples. With this open approach, not only the AMR genes associated with the known GMM constructs were retrieved, but also the *Bacillus*-specific *catA* gene associated with the novel GMM alpha-amylase2 construct (see Section 3.2.3.3), as well as several AMR genes associated with the unculturable *Bacillus* strains that contaminated the samples. Notably, the *catA* gene was not detected by our previously developed qPCR assay targeting a *cat* gene commonly present in vectors, which was found in an unauthorized GMM on at least one occasion [3]. The *cat* gene targeted in this qPCR assay originates from *S. aureus*, and shows only 42.8% sequence similarity at the nucleotide level with the *cat* gene indigenous to *Bacillus*, explaining why the latter did not produce a positive signal with this assay.

In addition to the more complete characterization of known GMM strains and constructs, the metagenomic approach also revealed the presence of several previously undetected *Bacillus* strains, and allowed for the discovery and characterization of a novel transgenic construct, GMM alpha-amylase2, which was shown to be integrated into the chromosome of its host *B. licheniformis*. The FE products contained up to three unculturable *Bacillus* strains: two *B. licheniformis* and one *B. amyloliquefaciens* strain(s) that were likely deliberately engineered to impair their ability to sporulate. Concerning the suspected artificial nature of the genetic modifications, the *sigF-spoIIAB* deletion is especially noteworthy. At the site of the deleted region, a short foreign sequence was detected (GACTCTAGAGGATCCCC, Figure S7), which was not present in strain ATCC9789. This 17 bp sequence is an exact match to the multiple cloning site (MCS) of plasmid pWH1520. In a recent study [50], this plasmid was employed as a vector for a CRISPR/Cas9 editing system for *B. licheniformis*. Zhou et al. cloned a CRISPR/Cas9 construct into the MCS of pWH1520 (Accession JC210951), resulting in the MCS ending up flanking the homologous repair template (HRT) of the CRISPR/Cas9 construct. This or a similar vector might therefore potentially have been used to construct the *sigF-spoIIAB* deletion by CRISPR/Cas9 editing, whereby a part of the flanking sequence of the HRT may have ended up in the genome of the *B. licheniformis* strain by accident, leading to the presence of a ‘trace’ sequence that could be detected in the resulting GMM strain. However, it should be emphasized that a 17 bp sequence is too short to unequivocally determine its origin, and whether the deletion was created with CRISPR/Cas9 or with another genetic engineering technique. 

Knock-out of sporulation genes is an established strategy in *Bacillus* producer strains, because it facilitates sterilization of the fermentation equipment, while it can also increase enzyme production yield [51]. A *Bacillus* strain unable to produce spores is unable to survive during long-term storage under unsuitable conditions for vegetative growth. Therefore, the presence of genetic modifications rendering the strains asporogenic could explain why they could not be isolated as viable strains, despite their high read abundance in some of the samples. 

With the aid of a high-depth sequencing short-read dataset for the Coobra sample, the GMM protease1 host strain could additionally be detected and partially characterized. This strain was not detected with the lower-depth datasets for the amylase samples Coobra, Stillspirits and Browin, which can on the one hand be attributed to its low read abundance, which may in turn be associated with its presence as spores, potentially reducing the efficiency of the DNA extraction, and on the other hand to the close relationship of *B. amyloliquefaciens* and *B. velezensis*, which likely caused the assemblies for both species to collapse and hide the presence of the strain present in the lowest abundance. With the continuing decrease in sequencing cost, this level of sequencing depth will become feasible, allowing to take full advantage of the power of metagenomics when in-depth metagenomic characterization of this type of complex datasets is envisaged.

Together, these results confirm that metagenomic analysis can partly bypass the need for cumbersome and often problematic isolation experiments, while additionally allowing to detect and characterize previously undetected constructs and strains, highlighting the potential of metagenomics and a hybrid assembly approach for the analysis of GMM-based products. 

A major obstacle for the detection of GMM by enforcement laboratories is that the dossiers submitted to EFSA by the manufacturers, providing detailed information concerning producer organisms and genetic modifications for the different FE products, are confidential. Therefore, even when a GMM is detected, the confidentiality of the data present in the dossier does not allow to verify by enforcement laboratories that the GMM described in the dossier is effectively the one present in the product sold on the market. Moreover, for one of the samples, the information that is publicly available was shown to be incorrect, i.e., the Pureferm FE is labeled to be produced with *B. subtilis* (Table 1), while our analysis demonstrated that it is in fact a *B. velezensis* strain. 

Due to this confidentiality and lack of information, it is difficult for enforcement laboratories to develop routine, targeted detection methods. Even if an open approach such as metagenomics was used, it is still difficult to draw definitive conclusions concerning the potential risks that are associated with these contaminations. The potential risk for spreading of AMR through horizontal gene transfer increases if AMR genes are located on mobile genetic elements, such as plasmids [52]. Although the GMM alpha-amylase1 construct most likely exists as a free high-copy plasmid, this could not be unequivocally established. Moreover, it was not possible to identify the host of this construct with full certainty, based on the available results. However, the amylase encoding gene in this construct originates from *B. amyloliquefaciens*, for which an unculturable strain was detected in the metagenomic data. The amylase encoding gene from the GMM alpha-amylase2 construct on the other hand was derived from *B. licheniformis* and was also shown to be associated with an unculturable *B. licheniformis* strain in the samples. Therefore, it could be deduced that the most likely host for GMM alpha-amylase1 is the unculturable *B. amyloliquefaciens* strain. To confirm this, prior isolation of the host strain would still be required, or alternatively the use of advanced analysis methods such as Hi-C, which relies on a sample pretreatment to cross-link genomic DNA regions in close proximity to one another, followed by NGS of linked DNA segments [53].

One of the most noteworthy findings from this study is that the samples were cross-contaminated with three different transgenic constructs. The cross-contaminations may have been caused by a common downstream processing line for both amylase and protease FE production, which is not sufficiently decontaminated between batches. Alternatively, the contaminations may originate from different manufacturers, and ended up together as a consequence of batch mixing. 

The use of GMM in food- and other industries has some undeniable advantages, and since microbial fermentation takes places in an enclosed environment, potential risks associated with the use of GMM can, at least in theory, be perfectly mitigated. However, these commercially available FE products contained a plethora of microbial contaminations, including, e.g., for the Coobra sample a viable GMM, a natural viable contamination, and DNA from three unculturable GMM, resulting in a combined significant AMR gene load. This signals a significant problem with the implementation of suitable containment procedures at the production facilities and poses a substantial potential public health risk, as the AMR genes could potentially spread into the environment, e.g., by horizontal transfer to gut microbiota and/or to pathogens after ingestion In turn, this emphasizes the need for more structural control procedures, to ensure the quality and safety of microbial fermentation products. The availability of detailed information concerning species, strain and genetic modifications of registered GMM to control enforcement laboratories would enable the development of targeted detection methods. In particular, the implementation of a GMM reference database, analogous to, e.g., the GMO database Nexplorer [54] or JRC GMO-Amplicons [55], would allow for the development of much more efficient NGS analysis pipelines. 

## Figures and Tables

**Table 1:** Overview of results of classical methods for characterization of food enzyme (FE) products, showing the qPCR results (average Cq of duplicate runs) for GMM transgenic constructs on the FE matrix, a description of the viable strains isolated from the FE matrix, and the associated RASFF identifiers.

Commercial FE Product (Supplier)	Labeled Enzymes	Labeled Producer Organism	Application	GMM Alpha-Amylase1 (Cq)	GMM Protease1 (Cq)	GMM Protease2 (Cq)	Viable Isolates	RASFF
Alpha-amylase enzyme 4 g (**Coobra** ^1^)	Alpha-amylase	Unknown	Distillery	18.1	19.7	-	*B. velezensis* GMM protease1 (previous study [8]); *B. licheniformis*, presumably a natural strain	RASFF2020.2582
Distiller’s Enzyme Alpha-Amylase (**Stillspirits** ^1^)	Alpha-amylase	Bacteria	Distillery, brewing	15.2	36.4	-	no viable *Bacillus* strains detected	RASFF2020.2579
Alpha-amylase 4 g (**Browin** ^1^)	Alpha-amylase	Unknown	Distillery	18.2	19.8	-	no viable *Bacillus* strains detected	RASFF2020.2577
**Pureferm**^1^ (The Alchemist’s Pantry)	Neutral protease	*B. subtilis*	Cereal based products	22.8	12	-	GMM protease1 (previous study [8]); *B. velezensis* ^2^,	RASFF2019.3332 [9]

^1^ Names in bold are used to indicate the samples throughout the manuscript. ^2^ Presumably GMM protease1 host strain from which plasmid with transgenic construct was lost due to absence of selection pressure.

**Table 2:** Metrics of hybrid metagenomic assemblies generated with OPERA-MS using a combination of short and long reads, and derived metagenomics assembled genomes (MAGs) in the FE samples.

Metagenome or MAG ^1^	Short-Read Coverage	Long-Read Coverage	Total Length (bp)	# Contigs	Longest Contig (bp)	Contig N50 (bp)	GC% ^2^	Completeness (%) ^2^	Taxonomic Classification ^2^
Coobra—metagenome			9,466,426	2532	986,809	344,089			
OPERA-MS									
MAG 1	439×	288×	4,204,618	15	781,319	438,830	46.1	98.96	*B. licheniformis*
MAG 2	35×	59×	2,493,221	7	986,809	409,104	46.3	37.93	*B. amyloliquefaciens*
MAG 3	52×	61×	1,336,662	46	344,089	228,463	45.9	29.73	*B. amyloliquefaciens*
MetaBAT2									
MAG 1			3,465,643	13	986,809	344,089	46.3	70.69	*B. amyloliquefaciens*
MAG 2			4,146,435	14	781,319	438,830	46.1	81.03	*B. licheniformis*
Stillspirits—metagenome			9,622,923	2878	1,248,082	316,451			
OPERA-MS									
MAG 1	40×	41×	1,727,558	53	344,009	189,153	46.0	38.63	*B. amyloliquefaciens*
MAG 2	30×	38×	2,089,578	6	540,913	410,461	46.3	37.93	*B. amyloliquefaciens*
MAG 3	346×	160×	4,161,286	15	1,248,082	438,492	46.1	98.96	*B. licheniformis*
MetaBAT2									
MAG 1			3,628,827	17	540,913	344,009	46.2	79.31	*B. amyloliquefaciens*
MAG 2			4,103,086	14	1,248,079	438,490	46.1	81.03	*B. licheniformis*
Browin—metagenome			9,744,356	7644	763,810	2805			
OPERA-MS									
MAG 1	675×	54×	4,071,927	25	763,810	438,830	46.1	98.13	*B. licheniformis*
MetaBAT2									
MAG 1			442,319	130	9850	3241	46.6	8.62	*B. amyloliquefaciens*
MAG 2			4,094,526	24	763,810	438,830	46.1	81.03	*B. licheniformis*
Pureferm—metagenome			9,651,356	3008	878,492	497,635			
OPERA-MS									
MAG 1	238×	415×	4,182,772	13	878,492	610,759	46.0	98.33	*B. velezensis*
MAG 2	24×	99×	4,046,983	26	763,807	438,887	46.1	96.34	*B. licheniformis*
MetaBAT2									
MAG 1			4,124,570	26	763,807	438,886	46.1	97.42	*B. licheniformis*
MAG 2			591,152	3	519,275	519,275	46.1	4.17	*B. velezensis*
MAG 3			2,915,775	6	878,492	703,595	45.9	68.42	*B. velezensis*
MAG 4			610,759	1	610,759	610,759	46.2	0.00	*B. velezensis* ^3^

^1^ For each metagenome, the MAGs directly outputted by OPERA-MS by a reference-based clustering (i.e., supervised) approach are shown, together with the average short-read and long-read coverage that was obtained for each MAG. MAGs obtained by an alternative unsupervised binning tool, Metabat2, are presented as well. Taxonomic classification was done with GTDB-Tk. ^2^ GC%, completeness, and taxonomic classification are only relevant for the MAGs and are therefore not indicated for the metagenomes.^3^ GTDB-Tk did not assign a taxonomic label to this MAG (because it was too incomplete). Blastn was used to get an indication of the taxonomic classification.

**Table 3:** Metrics of metagenomic assembly generated with OPERA-MS based on the super-high depth short-read dataset and the long-read dataset described previously, and derived metagenomics assembled genomes (MAGs) in the Coobra sample.

Metagenome or MAG ^1^	Short-Read Coverage	Long-Read Coverage	Total Length (bp)	# Contigs	Longest Contig (bp)	Contig N50 (bp)	GC%^1^	Completeness (%) ^2^	Taxonomic Classification ^2^
metagenome			16,336,231	10,892	839,123	30,525			
OPERA-MS									
MAG 1	292×	46×	2,402,950	5	839,123	598,116	46.3	41.38	*B. amyloliquefaciens*
MAG 2	293×	48×	2,145,402	179	265,216	81,504	45.7	37.41	*B. amyloliquefaciens*
MAG 3	3817×	286×	4,069,496	15	783,520	312,887	46.2	98.13	*B. licheniformis*
MetaBAT2									
MAG 1			3,613,817	14	839,122	503,407	46.3	79.31	*B. amyloliquefaciens*
MAG 2			1,772,681	318	37,470	5949	46.7	0.00	*B. velezensis*
MAG 3			214,405	29	31,143	12,839	35.7	0.00	*B. velezensis* ^3^
MAG 4			4,101,999	16	783,520	312,888	46.1	81.03	*B. licheniformis*

^1^ The MAGs directly outputted by OPERA-MS by a reference-based clustering (i.e., supervised) approach are shown, together with the average short-read and long-read coverage that was obtained for each MAG. MAGs obtained by an alternative unsupervised binning tool, Metabat2, are presented as well. Taxonomic classification was done with GTDB-Tk. ^2^ GC%, completeness, and taxonomic classification (done with GTDB-Tk) are only relevant for the MAGs and are therefore not indicated for the metagenomes. ^3^ GTDB-Tk did not assign a taxonomic label to this MAG (because it was too incomplete). Blastn was used to get an indication of the taxonomic classification.

**Table 4:** Overview of the contribution of different analysis approaches to the elucidation of the microbial composition of the FE sample Coobra.

Strain/GMM	qPCR	Microbial Isolation + WGS	Metagenomics
GMM protease1 ^1^	x	x	x
pUB110-protease1 transgenic construct (episomal plasmid)	x	x	x
*B. velezensis* viable—host strain		x	x
Viable *B. licheniformis* strain ^2^		x	
GMM alpha-amylase1	x		x
pUB110-amylase1 transgenic construct (episomal plasmid)	x		x
*B. amyloliquefaciens*—∆*sigK* unculturable, putative host strain			x
GMM alpha-amylase2			x
transgenic construct GMM amylase2 integrated in host chromosome			x
*B. licheniformis*—∆*sigF-spoIIAB* unculturable—host strain			x
*B. licheniformis*—∆*yqfD* unculturable—potential host strain			x

‘x’ indicates that the approach was able to detect the strain/construct. ^1^ The viable GMM protease1 (*B. velezensis*) strain and the transgenic construct it carries were characterized previously [8]. ^2^ A potential explanation for the absence of the viable *B. licheniformis* strain from the metagenomic assembly is given in Supplementary text S6.

## Data Availability

Raw data and assemblies were deposited in the European Nucleotide Archive under study accession number PRJEB53495.

## Supplementary Materials

The following supporting information can be downloaded at: https://www.mdpi.com/article/10.3390/life12121971/s1, Figure S1: Visualization of taxonomic classification of metagenomic data (short-reads). Figure S2: Part of the whole-genome alignment of the *B. licheniformis* OPERA-MS MAGs, a number of the *B. licheniformis* isolates from Coobra, and a selection of reference strains, centered on a genomic island, indicated in blue, that is shared by *B. licheniformis* ATCC 9789 and the *B. licheniformis* MAGs, but is absent from the reference strains, and the *B. licheniformis* isolates. Figure S3: Part of the whole-genome alignment of *B. licheniformis* OPERA-MS MAGs, *B. licheniformis* isolate (no. 9), and a selection of reference strains, centered on the *sigF and spoIIAB* gene. Figure S4: Alignment of raw long reads of A. The Coobra, and B. the Pureferm sample to reference *B. licheniformis* ATCC 9789, centered on the *sigF* and *spoIIAB* genes. Figure S5: Whole-genome alignment of *B. licheniformis* OPERA-MS MAGs, *B. licheniformis* isolates (only no. 9 shown), and a selection of reference strains, centered on the *yqfD* gene. Figure S6: Multiple sequence alignment (ClustalO 1.2.4) of Sanger sequencing result of PCR product targeting the 728 bp *yqfD* deletion in *B. licheniformis* strain A. Figure S7: Multiple sequence alignment (ClustalO 1.2.4) of Sanger sequencing result of PCR product targeting the 151 bp *sigF-spoIIAB* deletion in *B. licheniformis* strain B. Figure S8: Alignment of raw long reads of the Coobra sample to reference *B. licheniformis* ATCC 9789, centered on the *amyS* gene, visualized with IGV. Figure S9: Alignment of raw long reads of the Coobra sample to reference *B. licheniformis* ATCC 9789, centered on the *catA* gene (cds 2,725,109–2,725,759), visualized with IGV. Figure S10: Multiple sequence alignments (ClustalO 1.2.4) of Sanger sequencing results of PCR products targeting the unnatural associations due to the insertion of the GMM alpha-amylase2 construct (*catA*-*amyS*) in the *B. licheniformis* host genome. Table S1: Metrics of assemblies for the *Bacillus* isolates from the Coobra and Pureferm FE samples, together with the SNP addresses^1^ obtained with *B. licheniformis* ATCC 9789 and *B. velezensis* Pilsner1-2 as reference genomes for the *B. licheniformis* and *B. velezensis* isolates, respectively. Table S2: Key metrics for Illumina and ONT raw data. Table S3: Overview of contigs with (putative) extrachromosomal elements detected in the metagenomic hybrid assemblies, Table S4: Result of AMR gene detection on raw short-read data. Table S5: Result of AMR gene detection on raw long-read data. Table S6: Overview of deletions supported by the long reads as compared to the reference *B. licheniformis* ATCC9789, with gene name and annotation of strain ATCC9789. Table S7: Depth and breadth of coverage of short and long reads that map uniquely against reference genomes of the *Bacillus* species found in the metagenomic samples, as well as the two extrachromosomal elements; plasmid pFL7 of *B. licheniformis* and the putative prophage of *B. velezensis,* and the three transgenic constructs GMM protease1, pUB110-amylase, and GMM alpha-amylase2. Table S8: Metrics of metagenomic long-read assemblies generated with Canu, and derived metagenomics assembled genomes (MAGs), obtained with Metabat2. Table S9: Metrics of hybrid metagenomic assemblies from the mock metagenomic data sets (Supplementary text S6). Supplementary text S1: Characterization of viable strains isolated from samples Coobra and Pureferm, Supplementary text S2: The GMM alpha-amylase1 construct is likely carried on an episomal high-copy plasmid, Supplementary text S3: The *Bacillus* contaminations constitute a considerable AMR gene load in the FE samples, Supplementary text S4: The *B. licheniformis* MAG in the alpha-amylase samples Coobra, Stillspirits and Browin represents two closely related *B. licheniformis* strains with deletions affecting their sporulation ability, Supplementary text S5: Discovery and characterization of novel construct GMM alpha-amylase2, Supplementary text S6: The *B. velezensis* GMM protease1 genome likely collapses with that of the unculturable B. amyloliquefaciens (GMM alpha-amylase1) strain in all the assemblies, Supplementary text S7: Challenges and bottlenecks of the bioinformatics analysis. References [56,57,58,59,60,61,62] have been cited in Supplementary Materials.
