# Supplementary material for: Metagenomic Characterization of Multiple Genetically Modified Bacillus Contaminations in Commercial Microbial Fermentation Products
Source: Life (Basel). 2022 Nov 25;12(12):1971. doi: 10.3390/life12121971 (PMC9781105; doi:10.3390/life12121971)
Supplement: Supplementary file 1 [file life-12-01971-s001.zip › life-2029052-supplementary.pdf]

1. Supplementary Figures

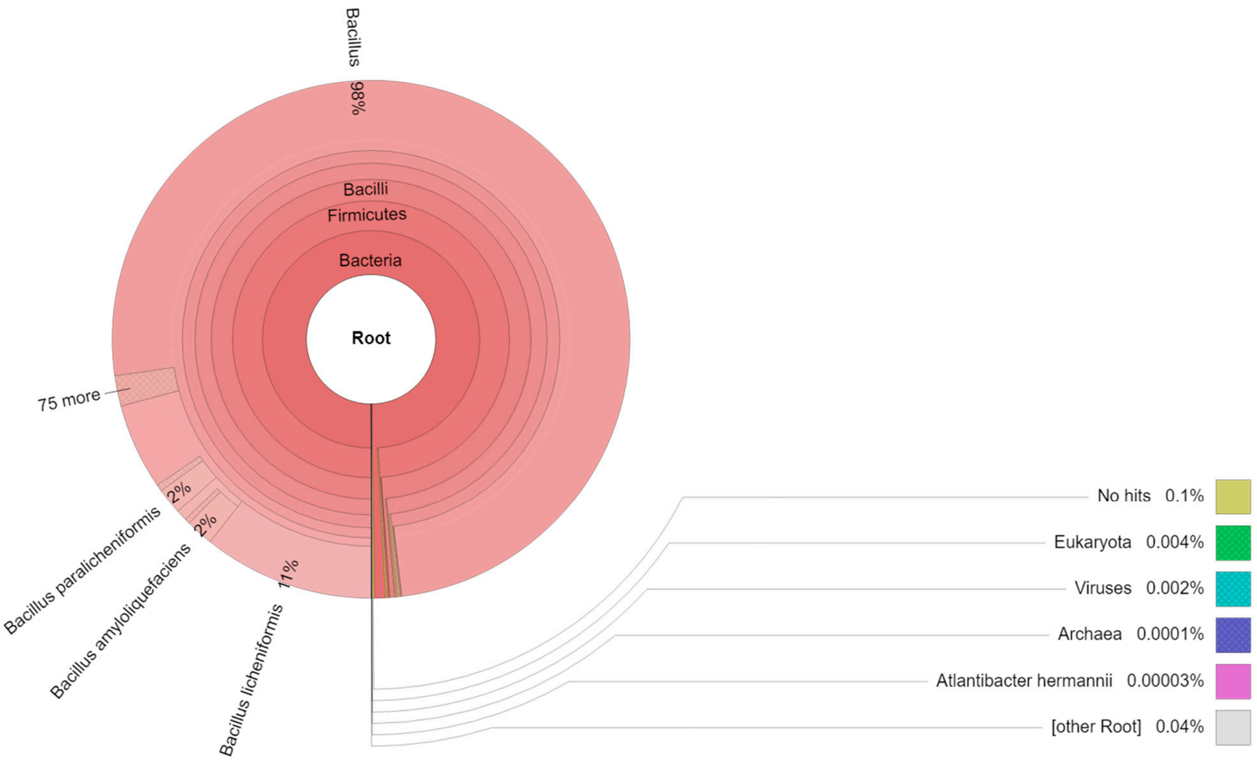

A.

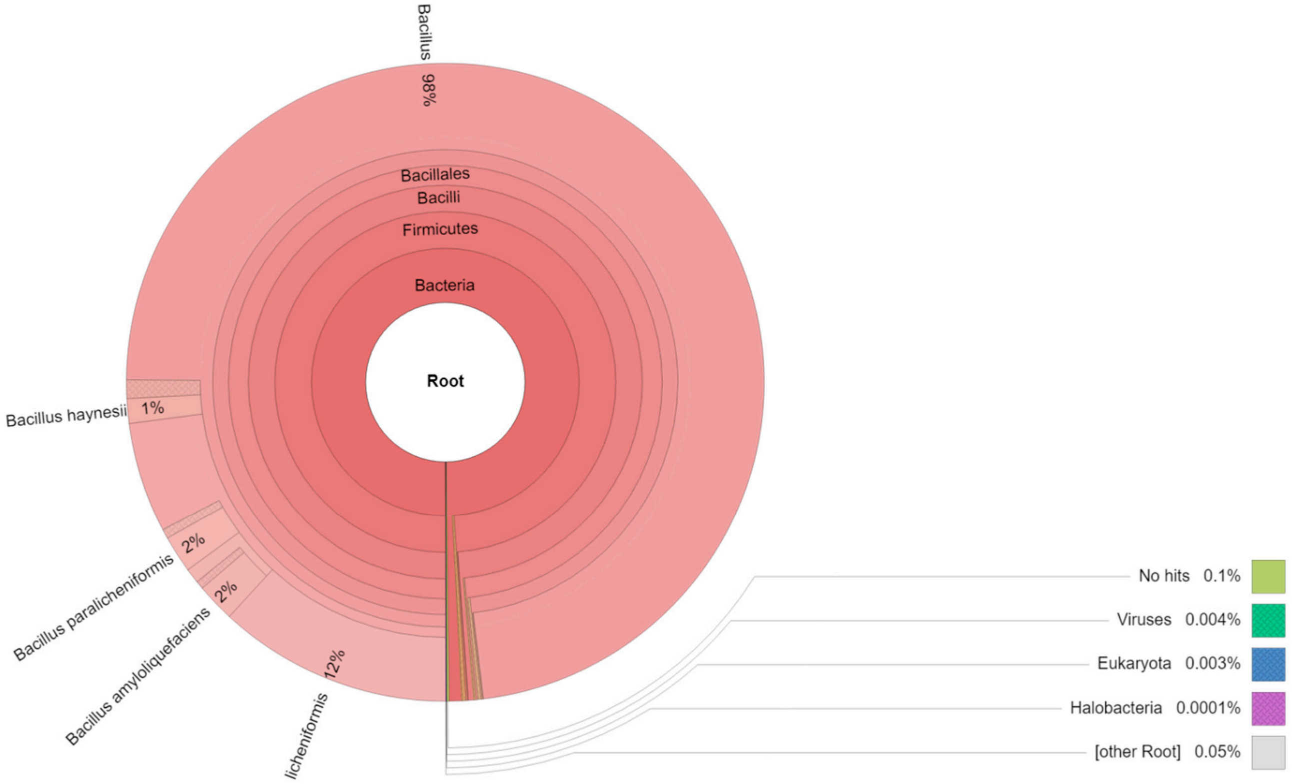

B.

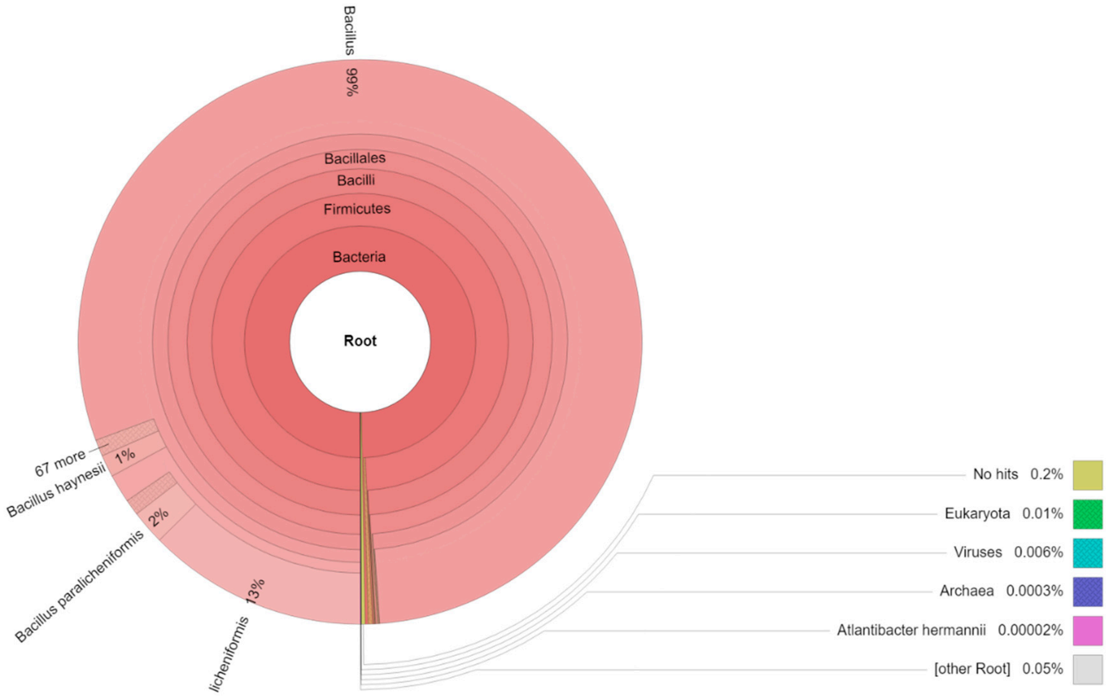

C.

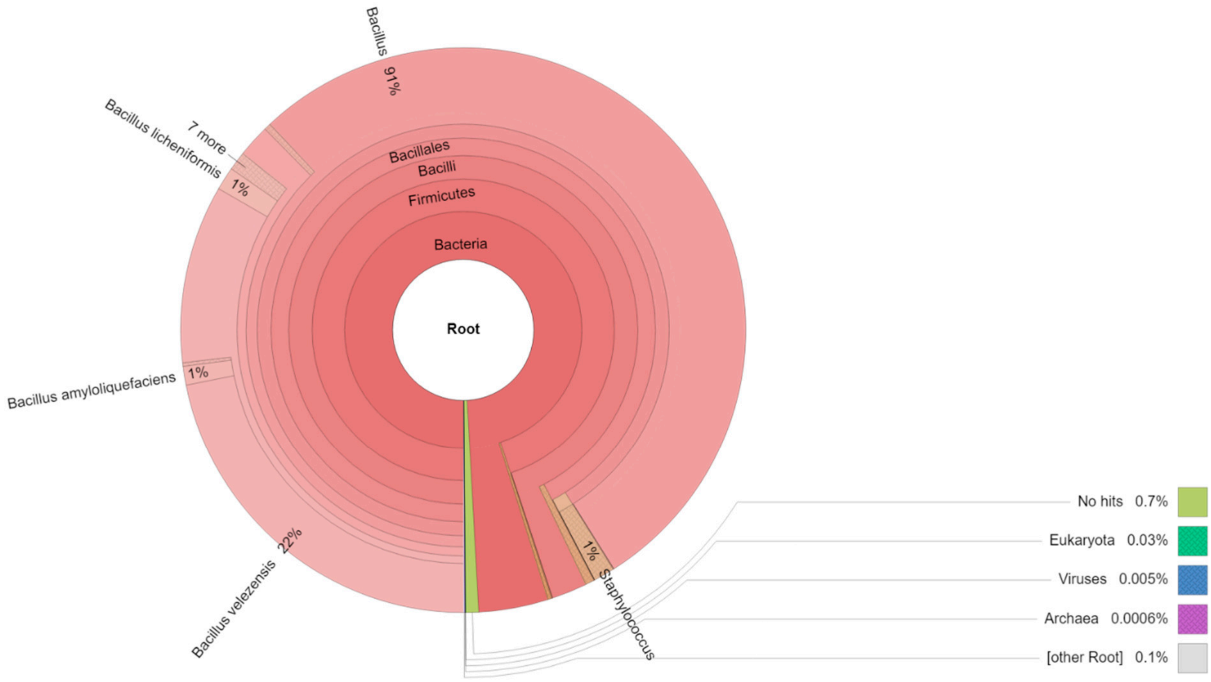

D.

Figure S1: Visualization of taxonomic classification of metagenomic data (short-reads).

A) Coobra (results for high-depth sequencing shown, 'normal-depth' results appear identical), B) Stillspirits, C) Browin, D) Pureferm. Figures rendered with Krona [34]. For all four FE samples, a large majority of the raw short reads were classified as *Bacillus*, whereas not a single other genus was present in abundances >1%, indicating a potentially significant contamination. A notable exception is the presence of reads classified as *Staphylococcus aureus*, which can be explained by the presence of pUB110 vector in the samples, which originates from *S. aureus* [46].

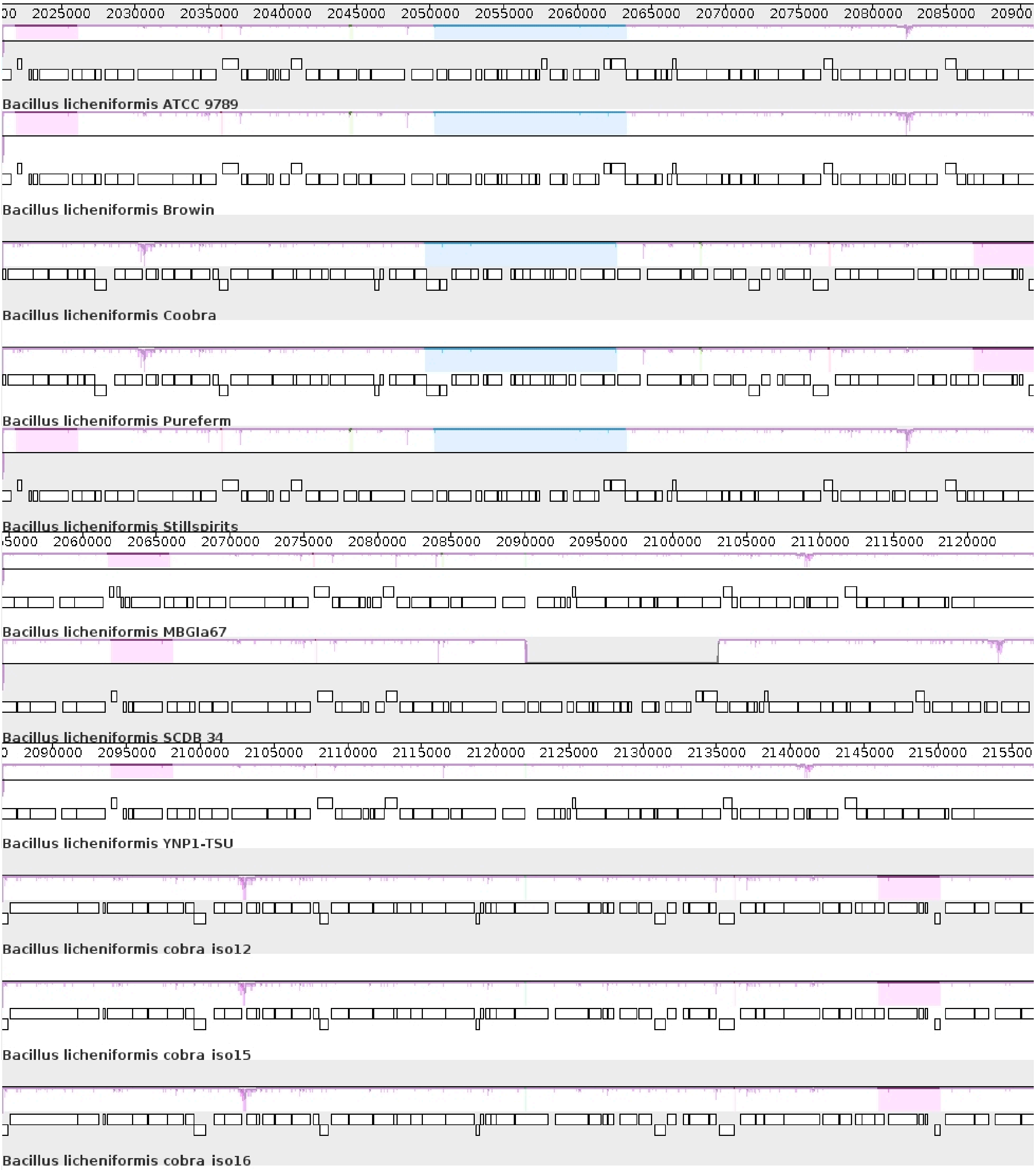

Figure S2: Part of the whole-genome alignment of the *B. licheniformis* OPERA-MS MAGs, a number of the *B. licheniformis* isolates from Coobra, and a selection of reference strains, centered on a genomic island, indicated in blue, that is shared by *B. licheniformis* ATCC 9789 and the *B. licheniformis* MAGs, but is absent from the reference strains, and the *B. licheniformis* isolates.

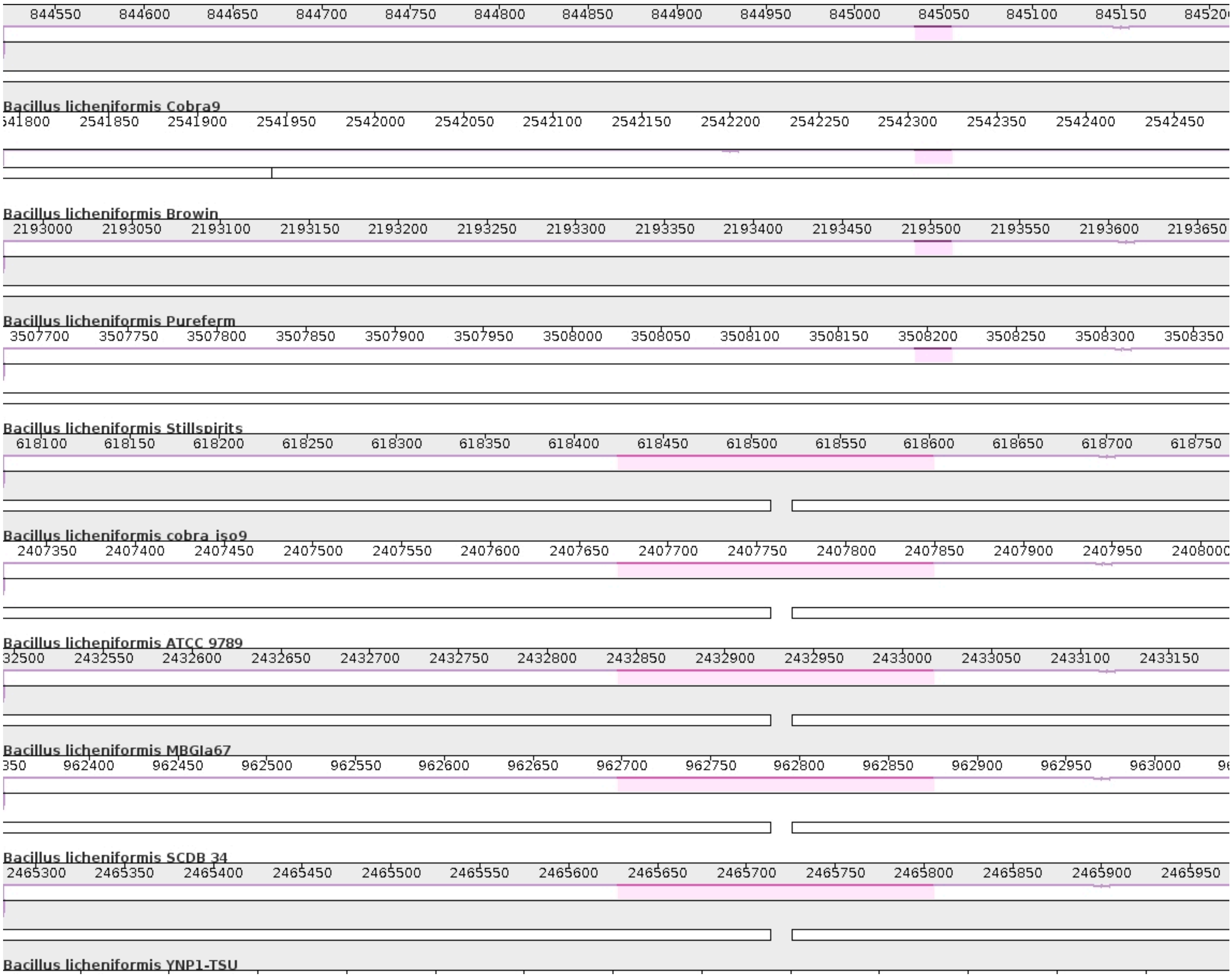

Figure S3: Part of the whole-genome alignment of *B. licheniformis* OPERA-MS MAGs, *B. licheniformis* isolate (no. 9), and a selection of reference strains, centered on the *sigF and spoIIAB* gene.

The colored tracks indicate the multiple sequence alignment, while the white bars below each alignment track represents the sequence annotation, with each bar representing a gene. The image shows a region of 174 bp in length on the alignment track, highlighted in pink, present in all the reference genomes, as well as in all the *B. licheniformis* isolates (only isolate 9 is shown), that is lacking from the genome assemblies of the unculturable *B. licheniformis* MAGs. This region spans the terminus of *sigF* (encoding sigma factor F) and start of *spoIIAB* (anti sigma factor F). The deletion causes the two genes in the *B. licheniformis* MAGs to become fused, according to the annotation track. Conversely, in the *B. licheniformis* MAGs a small region is highlighted in pink, which is a 17 bp foreign sequence (the putative crispr/Cas signature) that replaces the deleted chromosomal region, and is lacking in all the other genomes/assemblies (image created with Mauve Viewer).

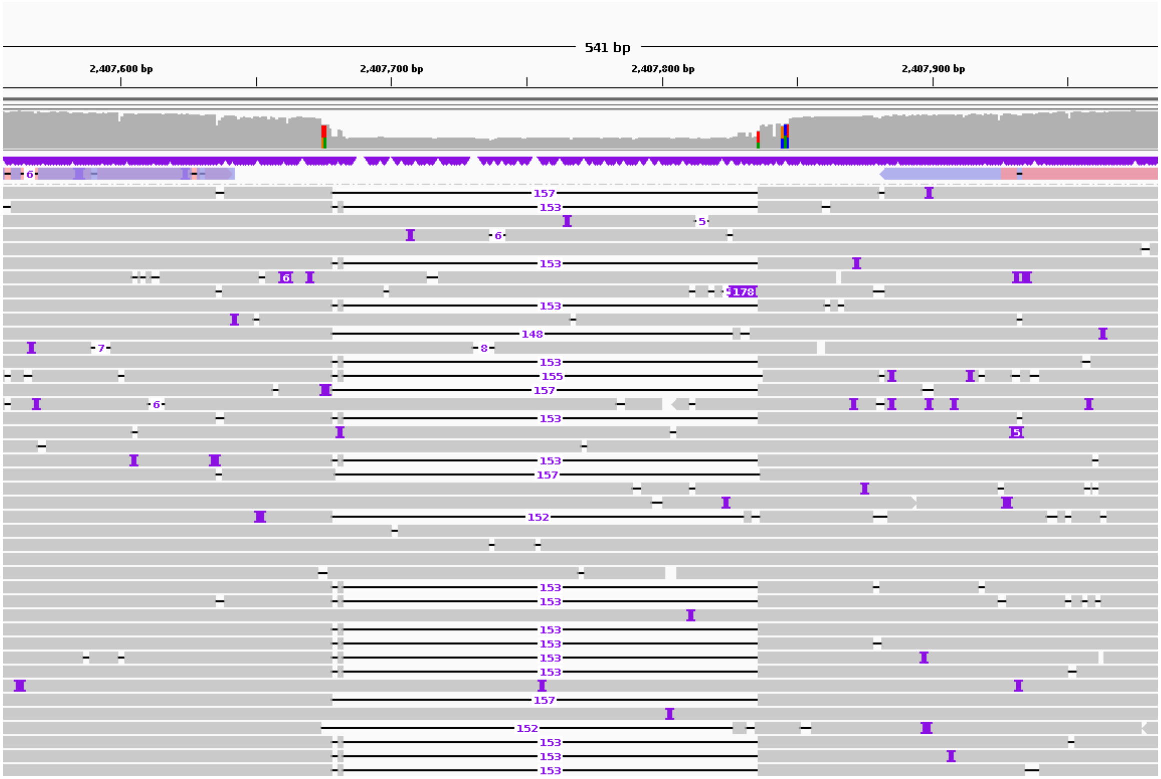

A.

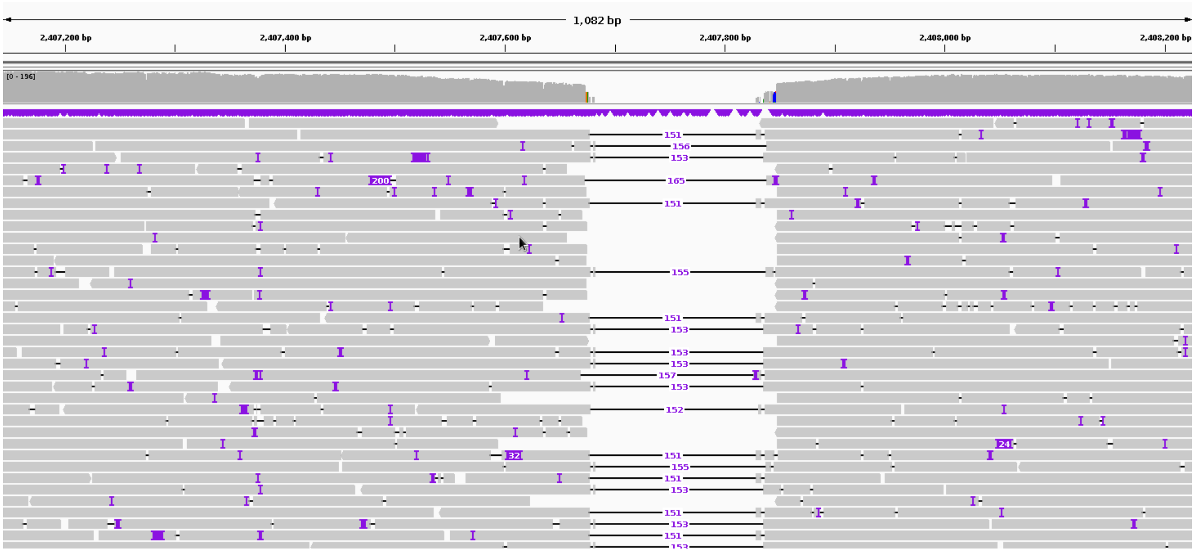

B.

Figure S4: Alignment of raw long reads of A. The Coobra, and B. the Pureferm sample to reference *B. licheniformis* ATCC 9789, centered on the *sigF* and *spoIIAB* genes.

At the site of the 151 bp deletion, coverage drops to zero for the Pureferm sample, indicating that in this Pureferm sample only a single *B. licheniformis* strain is present. In the Coobra sample however, the coverage also drops sharply but not to zero, as part of the reads provide coverage of the deletion site, supporting the presence of two strains; one that carries the deletion, and one that does not (image created with IGV).

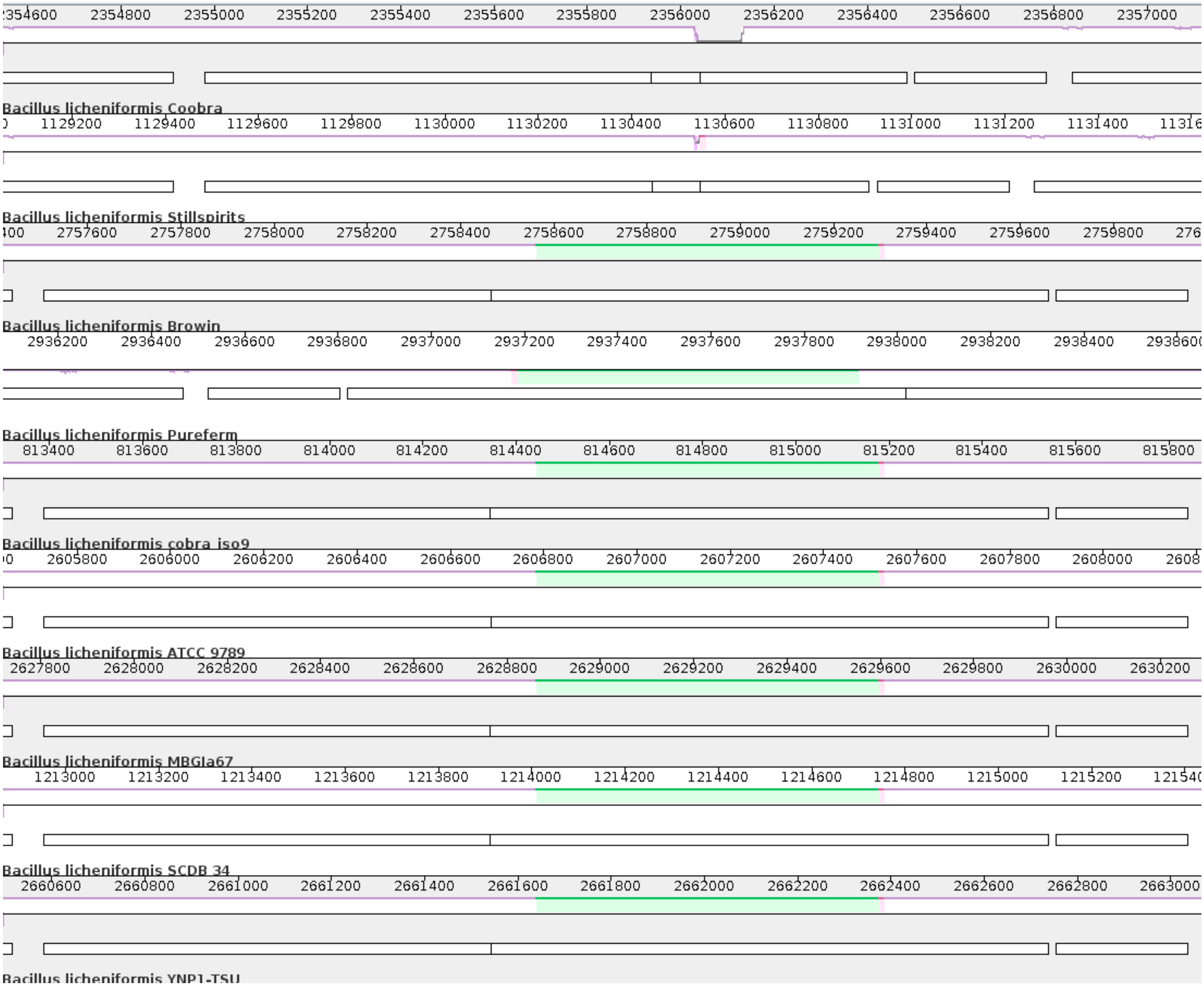

Figure S5: Whole-genome alignment of *B. licheniformis* OPERA-MS MAGs, *B. licheniformis* isolates (only no. 9 shown), and a selection of reference strains, centered on the *yqfD* gene.

The colored tracks indicate the multiple sequence alignment, while the white bars below each alignment track represents the sequence annotation, with each bar representing a gene. The light green shaded areas are shared among strain ATCC9789, the Coobra *B. licheniformis* isolates, the Browin and Pureferm MAGs, and the other reference strains, but are absent from the *B. licheniformis* MAGs of Coobra and Stillspirits. This area covers part of the CDS of *yqfD*, indicating the presence of a deletion (728 bp) in this gene in the assemblies of Coobra and Stillspirits. The short pink shaded areas flanking the blue zones are regions of shared sequence similarity between all the genomes included in the whole genome comparison (image created with Mauve Viewer).

```
reference      CTTCTGCTTTTTCGCCATCTTGATCGCATACAAGACATCGCGTTCGGATATCTCGATTCC     60
pureferm       -------------------------------------TCGCGTTCGGATATCTCGATTCC     23
cobra          ---------------------GATCGCATACAAGACATCGCGTTCGGATATCTCGATTCC     39
                                                    ***********************

reference      TTTGCGGATCAGATTTAGGAGAGAGGCAAGCAGGCTGTCCGCGATTTCAAACGTTTCTTC     120
pureferm       TTTGCGGATCAGATTTAGGAGAGAGGCAAGCAGGCTGTCCGCGATTTCAAACGTTTCTTC     83
cobra          TTTGCGGATCAGATTTAGGAGAGAGGCAAGCAGGCTGTCCGCGATTTCAAACGTTTCTTC     99
               ************************************************************

reference      ATCTCCTGTCACATACACGGTTTCTCCGCGCGTGACAATTGAAATGTTCAGCTCTTCCTC     180
pureferm       ATCTCCTGTCACATACACGGTTTCTCCGCGCGTGACAATTGAAATGTTCAGCTCTTCCTC     143
cobra          ATCTCCTGTCACATACACGGTTTCTCCGCGCGTGACAATTGAAATGTTCAGCTCTTCCTC     159
               ************************************************************

reference      CATCAACTTCAAATGGGAATCCTGGTTCCCAAACAGCGCTTGAGCCTCATTCGGACTTTC     240
pureferm       CATCAACTTCAAATGGGAATCCTGGTTCCCAAACAGCGCTTGAGCCTCATTCGGACTTTC     203
cobra          CATCAACTTCAAATGGGAATCCTGGTTCCCAAACAGCGCTTGAGCCTCATTCGGACTTTC     219
               ************************************************************

reference      CAGTTGCTGATGAATCGCAAGTAAGTGTTCTGTCATTCTTTAGTCTCCTGAACAATTGGT     300
pureferm       CAGTTGCTGATGAATCGCAAGTAAGTGTTCTGTCATTCTTTAGTCTCCTGAACAATTGGT     263
cobra          CAGTTGCTGATGAATCGCAAGTAAGTGTTCTGTCATTCTTTAGTCTCCTGAACAATTGGT     279
               ************************************************************

reference      GTTGTTTGAACAATGTTTTCAATAACCTGGTAAAGGATGATCAATTTAACTTTACCATTC     360
pureferm       GTTGTTTGAACAATGTTTTCAATAACCTGGTAAAGGATGATCAATTTAACTTTACCATTC     323
cobra          GTTGTTTGAACAATGTTTTCAATAACCTGGTAAAGGATGATCAATTTAACTTTACCATTC     339
               ************************************************************

reference      TCGCTCGTTTCGTGCAaaactttttcactgataatgttcccgtctctgccgatttttttc     420
pureferm       TCGCTCGTTTCGTGCAaaactttttcactgataatgttcccgtctctgccgatttttttc     383
cobra          TCGCTCGTTTCGTGCA--------------------------------------------     355
               ****************

reference      ctgatgtctctttttcccatttcgattccttcaagaactgcttctttttttcgagtacacc     480
pureferm       ctgatgtctctttttcccatttcgattccttcaagaactgcttctttttttcgagtacacc     443
cobra          ------------------------------------------------------------     355

reference      cttttgatttgttcgctctccctcatatgctccttttcataagcgacaggaagcttaaaa     540
pureferm       cttttgatttgttcgctctccctcatatgctccttttcataagcgacaggaagcttaaaa     503
cobra          ------------------------------------------------------------     355

reference      ttcataaaatgcagcgagtgtttttcggtctccgtcttcgggcgcgagaagtcttctttt     600
pureferm       ttcataaaatgcagcgagtgtttttcggtctccgtcttcgggcgcgagaagtcttctttt     563
cobra          ------------------------------------------------------------     355

reference      ttaaatgaaaagccccagatcggcacggaaaatgatccgagggatagcttgtgacttgtc     660
pureferm       ttaaatgaaaagccccagatcggcacggaaaatgatccgagggatagcttgtgacttgtc     623
cobra          ------------------------------------------------------------     355

reference      cttactttacccgtaaaaacgtcaaatgatgtctcaagaggaaccgttactgttgacttg     720
pureferm       cttactttacccgtaaaaacgtcaaatgatgtctcaagaggaaccgttactgttgacttg     683
cobra          ------------------------------------------------------------     355

reference      taccaggtttcaccatagattttccctttttgctccgacttttgcttttcctcttcgctt     780
pureferm       taccaggtttcaccatagattttccctttttgctccgacttttgcttttcctcttcgctt     743
cobra          ------------------------------------------------------------     355

reference      ccgatcagcccggaaacgagcatttgcccttttttcaacgtgctggttcaccgtgacgagc     840
pureferm       ccgatcagcccggaaacgagcatttgcccttttttcaacgtgctggttcaccgtgacgagc     803
cobra          ------------------------------------------------------------     355

reference      ggctcgccttttttccacgaacattttcgagatggtcgccccttttttggcgacgatgtgc     900
pureferm       ggctcgccttttttccacgaacattttcgagatggtcgccccttttttggcgacgatgtgc     863
cobra          ------------------------------------------------------------     355

reference      ctcggaccgatatatttttctttgtcaggttcattcttttcaacgactttcatgtgaagg     960
pureferm       ctcggaccgatatatttttctttgtcaggttcattcttttcaacgactttcatgtgaagg     923
cobra          ------------------------------------------------------------     355

reference      gcggtaccgtttaactcaatgcccacccaagtgatgttttcgacccgctttgtgagcgcc     1020
pureferm       gcggtaccgtttaactcaatgcccacccaagtgatgttttcgacccgctttgtgagcgcc     983
cobra          ------------------------------------------------------------     355

reference      tgctgaatttttttccggggtcagcattgaaaactgaaagcggccttttttgacgccgatt     1080
pureferm       tgctgaatttttttccggggtcagcattgaaaactgaaagcggccttttttgacgccgatt     1043
cobra          ------------------------------------------------------------     35

reference      tgatccaattgctgtctgatttgATGTTCTGTCTCTGGATTGGCTCCTGTAATATCAATT     1140
pureferm       tgatccaattgctgtctgatttgATGTTCTGTCTCTGGATTGGCTCCTGTAATATCAATT     1103
```

```
cobra           ------------------------------------------------------------        355

reference       ctcggaccgatatatttttctttgtcaggttcattcttttcaacgactttcatgtgaagg        960
pureferm        ctcggaccgatatatttttctttgtcaggttcattcttttcaacgactttcatgtgaagg        923
cobra           ------------------------------------------------------------        355

reference       gcggtaccgtttaactcaatgcccacccaagtgatgttttcgacccgctttgtgagcgcc        1020
pureferm        gcggtaccgtttaactcaatgcccacccaagtgatgttttcgacccgctttgtgagcgcc        983
cobra           ------------------------------------------------------------        355

reference       tgctgaatttttttccggggtcagcattgaaaactgaaagcggccttttttgacgccgatt       1080
pureferm        tgctgaatttttttccggggtcagcattgaaaactgaaagcggccttttttgacgccgatt       1043
cobra           ------------------------------------------------------------        35

reference       tgatccaattgctgtctgatttgATGTTCTGTCTCTGGATTGGCTCCTGTAATATCAATT        1140
pureferm        tgatccaattgctgtctgatttgATGTTCTGTCTCTGGATTGGCTCCTGTAATATCAATT        1103
cobra           -----------------------ATGTTCTGTCTCCGGATTGGCTCCTGTAATATCAATT        392
                                       ************ ************************

reference       TTCCAAAGCATGTTGGACAATAGGAACATGATGATAAAAAATGCAGCAACTCCAAAAGTG        1200
pureferm        TTCCAAAGCATGTTGGACAATAGGAACATGATGATAAAAAATGCAGCAACTCCAAAA---        1160
cobra           TTCCAAAGCATGTTGGACAATAGGAACATGATGATAAAAAATGCAGCAACTCCAAAAGTG        452
                *********************************************************

reference       AAGCCGCTATTCCGTTTAGACTTCTGCACGAGGAAAGG       1238
pureferm        --------------------------------------       1160
cobra           AAGCCGCTATTCCGTTTA--------------------       470
```

Figure S6: Multiple sequence alignment (ClustalO 1.2.4) of Sanger sequencing result of PCR product targeting the 728 bp *yqfD* deletion in *B. licheniformis* strain A.

The reference is the genome sequence of *B. licheniformis* strain ATCC 9789. Primers are indicated in bold, deletion in lowercase.

Asterisks indicate positions in the alignment that show sequence identity among all the sequences in the alignment.

```
reference      ATGCAGCCGATTTGAAAGAGGTCGTCAGGCTCATAGCCTCTGTTCAAAAAACGCTGAACG      60
pure           --------------------GTCGTCAGGCTCATAGCCTCTGTTCAAAAAACGCTGAACG      40
cobra          --------------------GTCGTCAGGCTCATAGCCTCTGTTCAAAAAACGCTGAACG      40
                                   ****************************************

reference      ACAGACCAAACAAGACGCATGTTTTTTTCTATGAGGAGGTCCCTTGCTTTTTGATCGCCG      120
pure           ACAGACCAAACAAGACGCATGTTTTTTTCTATGAGGAGGTCCCTTGCTTTTTGATCGCCG      100
cobra          ACAGACCAAACAAGACGCATGTTTTTTTCTATGAGGAGGTCCCTTGCTTTTTGATCGCCG      100
               ************************************************************

reference      ttctggctgtttttaatcagttctttcacttcatggtctttaagctgagtgttctggttt      180
pure           GACTCTAGAGGATCCCC-------------------------------------------      117
cobra          GACTCTAGAGGATCCCC-------------------------------------------      117


reference      tctttttaacctccacatccataacaaatctcccttaattgcaaagcgctttgcttttt      240
pure           ------------------------------------------------------------      117
cobra          ------------------------------------------------------------      117


reference      gataagtgctttgttaagtgtattgtggttcccatctcaggtgaggagtcgatCGAAATA      300
pure           -----------------------------------------------------CGAAATA      124
cobra          -----------------------------------------------------CGAAATA      124
                                                                    *******

reference      TCATCCATGAAATTTTCCATGATCGTAAAGCCCATTCCCGACCGCTCGAGTTCAGGCTTT      360
pure           TCATCCATGAAATTTTCCATGATCGTAAAGCCCATTCCCGACCGCTCGAGTTCAGGCTTT      184
cobra          TCATCCATGAAATTTTCCATGATCGTAAAGCCCATTCCCGACCGCTCGAGTTCAGGCTTT      184
               ************************************************************

reference      GTCGTGAACAGGGGCTGGCGCGCTTCTTCAAGATCAGGGATGCCGACTCCTTCGTCGCGG      420
pure           GTCGTGAACAGGGGCTGGCGCGCTTCTTCAAGATCAGGGATGCCGACTCCTTCGTCGCGG      244
cobra          GTCGTGAACAGGGGCTGGCGCGCTTCTTCAAGATCAGGGATGCCGACTCCTTCGTCGCGG      244
               ************************************************************

reference      ATCGTTAAATAGACAATATGGTCCTCGAGAGTGACGGAAATATATACGTTTCCCTGCCCT      480
pure           ATCGTTAAATAGACAATATGGTCCTCGAGAGTGACGGAAATATATAC-------------      291
cobra          ATCGTTAAATAGACAATATGGTCCTCGAGAGTGACGGAAATATATAC-------------      291
               ***********************************************

reference      GAGTTTT487
pure           -------291
cobra          -------291
```

Figure S7: Multiple sequence alignment (ClustalO 1.2.4) of Sanger sequencing result of PCR product targeting the 151 bp *sigF-spoIIAB* deletion in *B. licheniformis* strain B.

The reference is the genomic sequence of *B. licheniformis* strain ATCC 9789. Primers are indicated in bold, deletion in lowercase.

Asterisks indicate positions in the alignment that show sequence identity among all the sequences in the alignment.

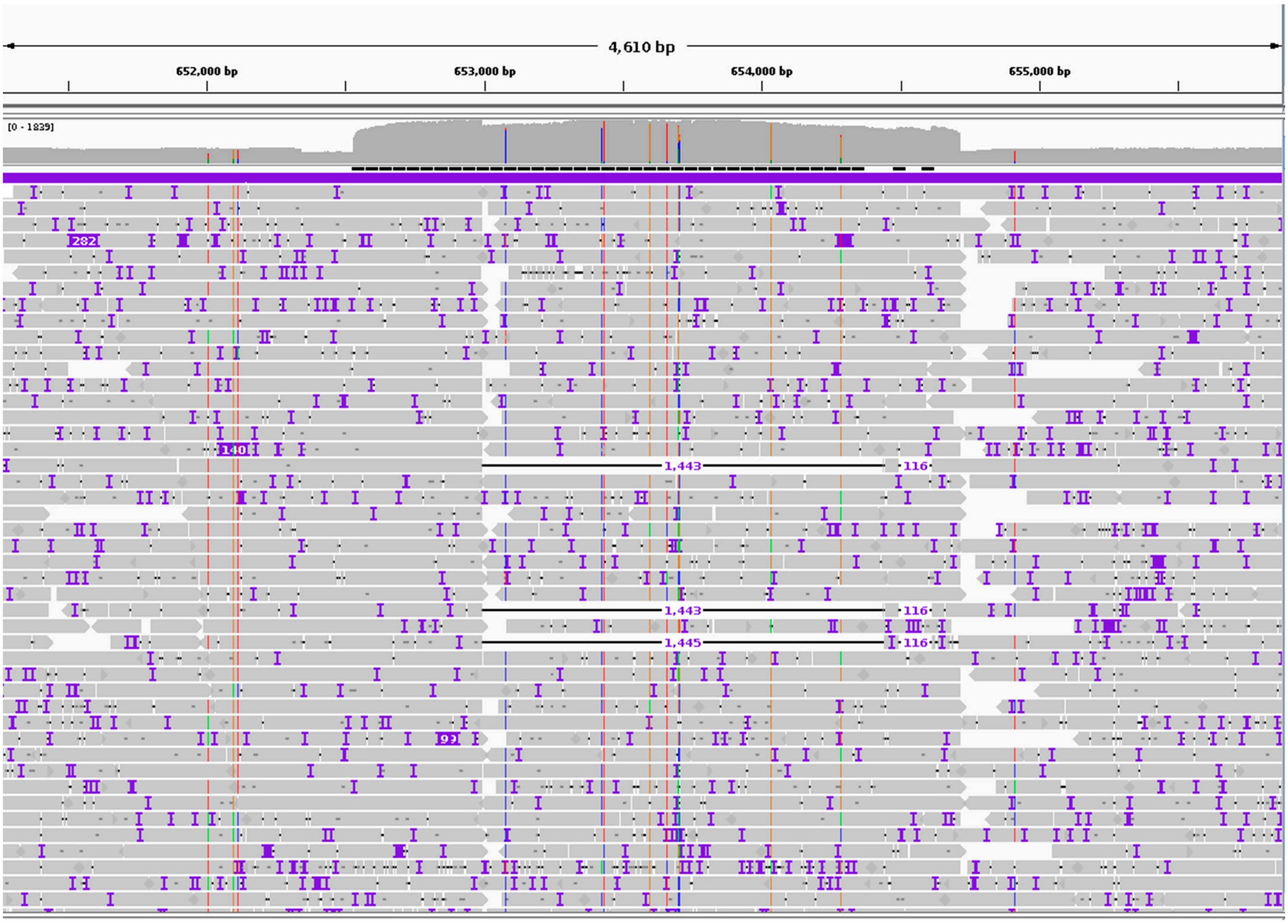

Figure S8: Alignment of raw long reads of the Coobra sample to reference *B. licheniformis* ATCC 9789, centered on the *amyS* gene, visualized with IGV.

The top track depicts the coverage, which can be observed to be much higher than in the surrounding regions. Note that reads spanning the *amyS* gene (nt 653,541-654,710 in reference) do not extend upstream or downstream of this region, indicating that the remainder of these reads does not align there, which is because they originate from the transgenic GMM alpha-amylase2 construct. On the other hand, a minority of the reads supports a deletion of *amyS*, as evident from reads with a gapped alignment, spanning the deleted region.

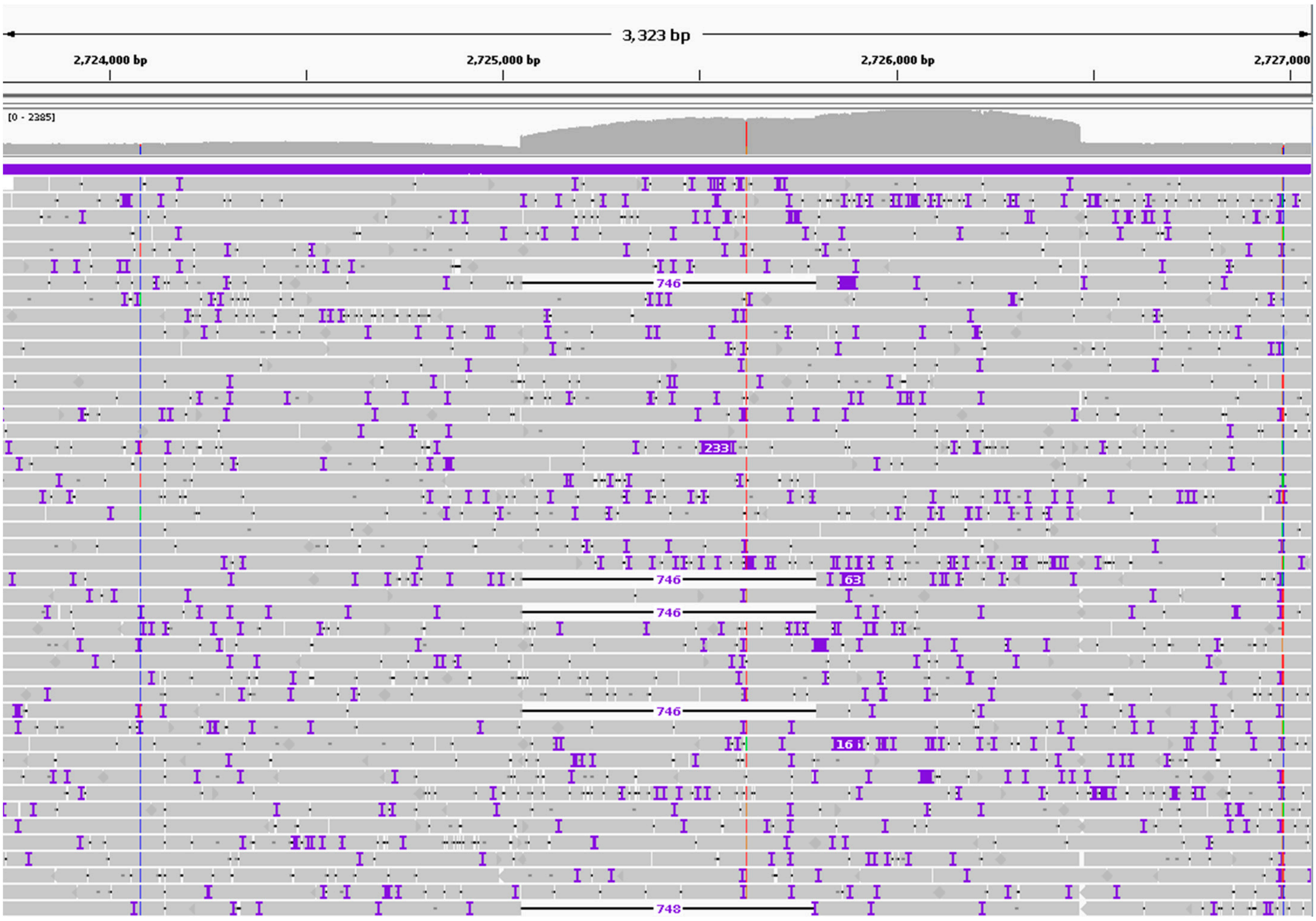

Figure S9: Alignment of raw long reads of the Coobra sample to reference *B. licheniformis* ATCC 9789, centered on the *catA* gene (cds 2,725,109-2,725,759) , visualized with IGV.

The top track depicts the coverage, which can be observed to be much higher than in the surrounding regions. At the same time, the alignment supports a 746 bp deletion, spanning the complete cds of *catA*, as evident from the reads with a gapped alignment, spanning the deleted region.

```
reference      GCGGGACTATGGATGTTTGTGATCGGCAGCTTTCAGCTGTTAATCAGGCCGACGATCCGT 60
pure           --------------------GATCGGCAGCTTTCAGCTGTTAATCAGGCCGACGATCCGT 40
cobra          ------------------------------------------ATCAGGCCGACGATCCGT 18
                                                         ******************

reference      TTGGTTCATGATTTTCACTACAGAAAGTTTGTTGAGAGCCGGCGTTCCAAATGCCGTGAG 120
pure           TTGGTTCATGATTTTCACTACAGAAAGTTTGTTGAGAGCCGGCGTTCCAAATGCCGTGAG 100
cobra          TTGGTTCATGATTTTCACTACAGAAAGTTTGTTGAGAGCCGGCGTTCCAAATGCCGTGAG 78
               ************************************************************

reference      TAAAATCACACAGCAGTGAGAGATGTTAcctgcttcactgattccggcaatattctcacc 180
pure           TAAAATCACACAGCAGTGAGAGATGTTAcctgcttcactgattccggcaatattctcacc 160
cobra          TAAAATCACACAGCAGTGAGAGATGTTAcctgcttcactgattccggcaatattctcacc 138
               ************************************************************

reference      ccatcctgtgttgtaaatgaattgtagcgcgtttcggaggtccaggcaacagtctc     236
pure           ccatcctgtgttgtaaatgaattgtagcgcgtttcg--------------------     196
cobra          ccatcctgtgttgtaaatgaattgtagcgcgtttcg--------------------     174
               ************************************
```

A.

```
reference      ggcagaatacatcctgcagaaaaggattgagaaagcggtcgatttgcttgctcattcaga       60
pure           -------------------aaaaggattgagaaagcggtcgatttgcttgctcattcaga       41
cobra          -------------------aaaaggattgagaaagcggtcgatttgcttgctcattcaga       41
                                  *****************************************

reference      acgatcgatcgcagaaatcgcttcaatggtaggcattgcgaatgcggaatat--------       112
pure           acgatcgatcgcagaaatcgcttcaatggtaggcattgcgaatgcggaatatCAAGCTTA       101
cobra          acgatcgatcgcagaaatcgcttcaatggtaggcattgcgaatgcggaatatCAAGCTTA       101
               ****************************************************

reference      ----------------------GATCACCCGCGATACCGTCATTTTCGACACATTTGCTT       150
pure           TCGGGCCGCTCTAGAACTAGTGGATCACCCGCGATACCGTCATTTTCGACACATTTGCTT       161
cobra          TCGGGCCGCTCTAGAACTAGTGGATCACCCGCGATACCGTCATTTTCGACACATTTGCTT       161
                                     **************************************

reference      TCTTTGCTACATCAGATAACGTTGCCATTTCATCCCCGCCTTACCTATGCGATTCAAACT       210
pure           TCTTTGCTACATCAGATAACGTTGCCATTTCATCCCCGCCTTACCTATGCGATTCAAACT       221
cobra          TCTTTGCTACATCAGATAACGTTGCCATTTCATCCCCGCCTTACCTATGCGATTCAAACT       221
               ************************************************************

reference      GTCAGCAAGTCCTTCCTGAGGGCTGATGACACTTTG 246
pure           GTCAGCAAGTCCTTCC-------------------- 237
cobra          GTCAGCAAGTCCTTCC-------------------- 237
               ****************
```

B.

```
reference      CTGCGGACGTTGCATAAATATCGTGCATTAAGACGATTTTTCCGTCTCCCGCATGGCTCA 60
pure           --------------------TCGTGCATTAAGACGATTTTTCCGTCTCCCGCATGGCTCA 40
cobra          --------------------TCGTGCATTAAGACGATTTTTCCGTCTCCCGCATGGCTCA 40
                                   ****************************************

reference      TGACATGATTGACAATCTTTTGCTTATTTTTGTACTTCCAATCTTCCGGATCAACATCCC 120
pure           TGACATGATTGACAATCTTTTGCTTATTTTTGTACTTCCAATCTTCCGGATCAACATCCC 100
cobra          TGACATGATTGACAATCTTTTGCTTATTTTTGTACTTCCAATCTTCCGGATCAACATCCC 100
               ************************************************************

reference      ACAATGAAACCTTCAGATTGGAAAGCGAGCGGACGGAATCATTGATCCCGCCGTATGGAG 180
pure           ACAATGAAACCTTCAGATTGGAAAGCGAGCGGACGGAATCATTGATCCCGCCGTATGGAG 160
cobra          ACAATGAAACCTTCAGATTGGAAAGCGAGCGGACGGAATCATTGATCCCGCCGTATGGAG 160
               ************************************************************

reference      GACGCAAGTGTAC-------------gttaagagacccgctaagaagtacatataaaaaa 227
pure           GACGCAAGTGTACGACCTCGAGGGGGgttaagagacccgctaagaagtacatataaaaaa 220
cobra          GACGCAAGTGTACGACCTCGAGGGGGgttaagagacccgctaagaagtacatataaaaaa 220
               *************             **********************************

reference      ggccgaatcatatcggcctttcctctgtcacacaagccactcctcacaatcggcggcaag 287
pure           ggccgaatcatatcggcctttcctctgtcacacaagccactcctcacaatcggcggcaag 280
cobra          ggccgaatcatatcggcctttcctctgtcacacaagccactcctcacaatcggcggcaag 280
               ************************************************************

reference      ccgttccaactcgtttataaaagcgccggcatgatagccgtcacacactgcat        340
pure           ccgttccaactcgtttataaaagcgccggcatg--------------------        313
cobra          ccgttccaactcgtttataaaagcgccggcatg--------------------        313
               *********************************
```

C.

```
reference      ggcagaatacatcctgcagaaaaggattgagaaagcggtcgatttgcttgctcattcaga 60
pureferm       -------------------aaaaggattgagaaagcggtcgatttgcttgctcattcaga 41
cobra          -------------------aaaaggattgagaaagcggtcgatttgcttgctcattcaga 41
                                  *****************************************

reference      acgatcgatcgcagaaatcgcttcaatggtaggcattgcgaatgcggaatatTTTGCCTC 120
pureferm       acgatcgatcgcagaaatcgcttcaatggtaggcattgcgaatgcggaatatTTTGCCTC 101
cobra          acgatcgatcgcagaaatcgcttcaatggtaggcattgcgaatgcggaatatTTTGCCTC 101
               ************************************************************

reference      TTTGTTTAAAAAGAAGACTGGGCAATCGCCGACGTCGTACCGGCAAATGAAACGGAGGTA 180
pureferm       TTTGTTTAAAAAGAAGACTGGGCAATCGCCGACGTCGTACCGGCAAATGAAACGGAGGTA 161
cobra          TTTGTTTAAAAAGAAGACTGGGCAATCGCCGACGTCGTACCGGCAAATGAAACGGAGGTA 161
               ************************************************************

reference      AACATATGGACGATCAAATCGTATACTGGCGTACGCTTATTTGCCGAGGTTGGCAGATTC 240
pureferm       AACATATGGACGATCAAATCGTATACTGGCGTACGCTTATTTGCCGAGGTTGGCAGAT-C 220
cobra          AACATATGGACGATCAAATCGTATACTGGCGTACGCTTATTTGCCGAGGTTGGCAGATTC 221
               ********************************************************** *

reference      ACATTGGGGCGACTGCCCGCGGGCTCTGTTTTACAGGGGGATGGAATCAA 290
pureferm       ACAT-GGGGCGACTGCCCGC------------------------------- 239
cobra          ACATTGGGGCGACTGCCCGCGGGCTCTGTT-------------------- 251
               **** ***************
```

D.

Figure S10: Multiple sequence alignments (ClustalO 1.2.4) of Sanger sequencing results of PCR products targeting the unnatural associations due to the insertion of the GMM alpha-amylase2 construct (*catA-amyS*) in the *B. licheniformis* host genome.

The numbering of the alignments corresponds to the red bars in Figure 2. The reference is a construction of the unnatural associations composed of the genomic sequence of *B. licheniformis* strain ATCC 9789. Primers are indicated in bold. Asterisks indicate positions in the alignment that show sequence identity among all the sequences in the alignment. A) Alignment region A: uppercase sequence corresponds to upstream region of *catA* in chromosome, lowercase sequence corresponds to downstream

region of *catA* in chromosome, B) Alignment region B: uppercase sequence corresponds to downstream region of *catA* in chromosome, lowercase sequence corresponds to *amyS*, C) Alignment region C: uppercase sequence corresponds to *amyS*, lowercase sequence corresponds to *catA*, D) Alignment region D: lowercase sequence corresponds to *catA*, uppercase sequence corresponds to downstream region of *catA* in chromosome

2. Supplementary Tables

Table S1: Metrics of assemblies for the *Bacillus* isolates from the Coobra and Pureferm FE samples, together with the SNP addresses[1] obtained with *B. licheniformis* ATCC 9789 and *B. velezensis* Pilsner1-2 as reference genomes for the *B. licheniformis* and *B. velezensis* isolates, respectively.

Sample/Isolate	**No. read pairs**	**# contigs**	**Longest contig (bp)**	**N50 (bp)**	**GC%**	**Total length (bp)**	**SNP address[1]**
**Coobra**							
2	540,522	29	841,559	449,313	46.21	4,153,643	28.28.30.30.31.32.33
3	649,476	42	415,891	182,134	46.26	4,122,964	28.28.30.30.31.32.33
4	680,915	72	317,111	132,179	46.36	4,106,118	28.28.30.30.31.32.33
6	659,874	64	489,729	128,657	46.31	4,109,150	28.28.30.30.31.32.33
7	741,303	55	524,562	145,031	46.29	4,115,715	28.28.30.30.31.32.33
8	555,871	25	1,171,299	841,559	46.21	4,153,936	28.28.30.30.31.32.33
9	565,438	28	1,171,341	841,559	46.21	4,154,394	28.28.30.30.31.32.33
12	433,563	27	863,890	709,205	46.21	4,153,584	28.28.30.30.31.32.33
15	710,758	40	523,604	221,882	46.26	4,134,200	28.28.30.30.31.32.33
16	539,197	31	824,599	438,717	46.22	4,153,669	28.28.30.30.31.32.33
**Pureferm**							
2	672,860	76	634,985	308,811	45.98	4,249,765	1.1.1.1.1.18.20
4	632,130	87	634,985	417,989	45.97	4,242,259	1.1.1.1.1.4.21
11	744,848	76	635,039	417,989	45.98	4,245,341	1.1.1.1.1.4.19

[1]The 7-number SNP addresses provide an isolate level hierarchical clustering nomenclature, whereby isolates sharing an increasing amount of SNPs, are increasingly more closely related. For example, for a group of isolates sharing only the first SNP address number, each isolate in the group is less than 250 SNPs away from at least one other isolate in the group. Likewise, the SNP thresholds are 100, 50, 25, 10, 5 and 0 for the second to last SNP address numbers, respectively. The SNP addresses of the references are set to 1.1.1.1.1.1.1., and the SNP addresses of the two GMM protease1 strains previously isolated from Pureferm were determined to be 1.1.1.1.1.4.5 and 1.1.1.1.3.3.4 [8].

Table S2: Key metrics for Illumina and ONT raw data.

Sample	Short reads (raw)	Long reads (raw)			
	No read pairs	Read length N50	No reads	No bp	Median read quality
Coobra 'normal depth'[1]	4,528,127	902	5,429,075	3,955,456,047	12.6
Coobra – 'high depth'[1]	40,185,924				
Stillspirits	3,620,680	968	2,355,810	1,856,931,199	12.7
Browin	6,379,179	670	5,143,670	2,791,974,285	12.4
Pureferm	2,404,857	677	13,676,337	8,050,723,605	14.1

[1] The 'high depth' short-read dataset of the Coobra sample is composed of the data resulting from an entire MiSeq run devoted to the Coobra sample, combined with the 'normal depth' Coobra dataset.

Table S3: Overview of contigs with (putative) extrachromosomal elements detected in the metagenomic hybrid assemblies

	Reference (name, description, reference, and length)	Stillspirits	Browin	Pureferm	Coobra 'normal depth'	Coobra 'high depth'
**construct GMM alpha-amylase1**	GMM alpha-amylase1 plasmid sequence derived from Coobra metagenomic assembly by trimming sequence duplication at ends (this study), 6,814 bp	1 contig of 6,895 bp, 100% identical match to full length of reference	1 contig of 6,847 bp, almost 100% identical match to full length of reference	1 contig of 2,350 bp, bp 1-150 matches pUB110, bp 151-2,350 matches the recombinant insert of the amylase construct	1 contig of 6,895 bp (used to derive sequence of reference)	1 contig of 6,895 bp
**construct GMM protease1**	GMM protease1 plasmid from *B. velezensis* isolate [5], Accession OU015425, 6,756 bp	1 contig of 2,547 bp, bp 1-80 and 2467-2547 match pUB110, intermediate sequence matches the recombinant insert of the protease construct	1 contig of 2,547 bp, bp 1-80 and 2467-2547 match pUB110, intermediate sequence matches the recombinant insert of the protease construct	1 contig of 610,759 bp, complete reference is present, assembled as part of the *B. velezensis* MAG chromosome	1 contig of 2,547 bp, bp 1-80 and 2467-2547 match pUB110, intermediate sequence matches the recombinant insert of the protease construct	1 contig of 2,547 bp, bp 1-80 and 2467-2547 match pUB110, intermediate sequence matches the recombinant insert of the protease construct
**prophage *B. velezensis***	Putative prophage from *B. velezensis* isolate [5], Accession OU015426, 13,899 bp	11 contigs, almost 100% identical to, and together covering 53% of reference	11 contigs, almost 100% identical to, and together covering 39% of reference	1 contig of 13,873 bp, 100% identical match to almost full length of reference	15 contigs, almost 100% identical to, and together covering 56% of reference	1 contig of 14,002 bp, 100% identical match to full length of reference
**Plasmid pFL7[1]**	pFL7, cryptic plasmid from *B. licheniformis*, Accession AJ577855, 7,853 bp	1 contig of 7,934 bp, almost 100% identical match to full length of reference	1 contig of 7,934 bp, almost 100% identical match to full length of reference	Not detected	1 contig of 7,934 bp, almost 100% identical match to full length of reference	1 contig of 7,934 bp, almost 100% identical match to full length of reference

[1]The Coobra, Stillspirits, and Browin assemblies displayed contigs that were an almost 100% identical full-length match to the natural cryptic plasmid pFL7 (Accession AJ577855). Literature reports the natural host of this plasmid being a *B. licheniformis* strain [56]. pFL7 was not detected in the Pureferm sample, nor in the *B. licheniformis* isolate assemblies, indicating that the unculturable *B. licheniformis* strain A is most likely its host.

Table S4: Result of AMR gene detection on raw short-read data.

Sample	Locus	Length	% Identity	% Covered	Depth	Antibiotic(s)	Accession
**Coobra – normal depth**	*clbA*	1050	93.05	100.00	25	Lincosamide;Macrolide;Streptogramin	NG_062350.1
	blaP	924	100.00	100.00	438	Beta-Lactam	NG_051162.1
	erm(D)	864	100.00	100.00	7	Macrolide	NG_047818.1
	ant(4')-Ia	762	100.00	100.00	5,536	Kanamycin;Tobramycin	NG_047373.1
	bleO	405	100.00	100.00	4,911	Bleomycin	NG_047557.1
	catA	651	100.00	100.00	994	Chloramphenicol	CP023729.1:2725109-2725759
**Coobra – high depth**	*clbA*	1050	93.05	100.00	241	Lincosamide;Macrolide;Streptogramin	NG_062350.1
	blaP	924	100.00	100.00	3,705	Beta-Lactam	NG_051162.1
	erm(D)	864	100.00	100.00	67	Macrolide	NG_047818.1
	ant(4')-Ia	762	100.00	100.00	48,765	Kanamycin;Tobramycin	NG_047373.1
	bleO	405	100.00	100.00	42,689	Bleomycin	NG_047557.1
	catA	651	100.00	100.00	9,008	Chloramphenicol	CP023729.1:2725109-2725759
**Stillspirits**	*clbA*	1050	93.05	100.00	28	Lincosamide;Macrolide;Streptogramin	NG_062350.1
	blaP	924	100.00	100.00	363	Beta-Lactam	NG_051162.1
	erm(D)	864	100.00	100.00	7	Macrolide	NG_047818.1
	ant(4')-Ia	762	100.00	100.00	4,888	Kanamycin;Tobramycin	NG_047373.1
	bleO	405	100.00	100.00	4,242	Bleomycin	NG_047557.1
	catA	651	100.00	100.00	903	Chloramphenicol	CP023729.1:2725109-2725759
**Browin**	*blaP*	924	100.00	100.00	703	Beta-Lactam	NG_051162.1
	erm(D)	864	100.00	100.00	15	Macrolide	NG_047818.1
	ant(4')-Ia	762	100.00	100.00	877	Kanamycin;Tobramycin	NG_047373.1
	bleO	405	100.00	100.00	813	Bleomycin	NG_047557.1
	catA	651	100.00	100.00	2,073	Chloramphenicol	CP023729.1:2725109-2725759
**Pureferm**	*blaP*	924	100.00	100.00	20	Beta-Lactam	NG_051162.1
	ant(4')-Ia	762	100.00	100.00	21,485	Kanamycin;Tobramycin	NG_047373.1
	bleO	405	100.00	100.00	20,243	Bleomycin	NG_047557.1
	catA	651	100.00	100.00	194	Chloramphenicol	CP023729.1:2725109-2725759

Table S5: Result of AMR gene detection on raw long-read data.

Sample	Locus	Length	% Identity	% Covered	Depth	Antibiotic(s)	Accession
**Coobra**	*clbA*	1050	93.05	100.00	23	Lincosamide;Macrolide;Streptogramin	NG_062350.1
	blaP	924	100.00	100.00	634	Beta-Lactam	NG_051162.1
	erm(D)	864	100.00	100.00	20	Macrolide	NG_047818.1
	ant(4')-Ia	762	100.00	100.00	4,216	Kanamycin;Tobramycin	NG_047373.1
	bleO	405	100.00	100.00	4,331	Bleomycin	NG_047557.1
	catA	651	100.00	100.00	1,828	Chloramphenicol	CP023729.1:2725109-2725759
**Stillspirits**	*clbA*	1050	93.140	100.00	13	Lincosamide;Macrolide;Streptogramin	NG_062350.1
	blaP	924	100.00	100.00	312	Beta-Lactam	NG_051162.1
	erm(D)	864	100.00	100.00	11	Macrolide	NG_047818.1
	ant(4')-Ia	762	100.00	100.00	2,043	Kanamycin;Tobramycin	NG_047373.1
	bleO	405	100.00	100.00	2,045	Bleomycin	NG_047557.1
	catA	651	100.00	100.00	943	Chloramphenicol	CP023729.1:2725109-2725759
**Browin**	*clbA*	1050	90.38	103.24	2	Lincosamide;Macrolide;Streptogramin	NG_062350.1
	blaP	924	100.00	100.00	418	Beta-Lactam	NG_051162.1
	erm(D)	864	99.88	99.88	11	Macrolide	NG_047818.1
	ant(4')-Ia	762	100.00	100.00	307	Kanamycin;Tobramycin	NG_047373.1
	bleO	405	100.00	100.00	422	Bleomycin	NG_047557.1
	catA	651	100.00	100.00	1,392	Chloramphenicol	CP023729.1:2725109-2725759
**Pureferm**	*blaP*	924	100.00	100.00	172	Beta-Lactam	NG_051162.1
	aph(3')-Ia	816	99.88	100.12	5	Kanamycin	NG_047430.1
	ant(4')-Ia	762	100.00	100.39	66,398	Kanamycin;Tobramycin	NG_047373.1
	bleO	405	100.00	100.00	71,379	Bleomycin	NG_047557.1
	catA	651	100.00	100.00	1,446	Chloramphenicol	CP023729.1:2725109-2725759

Table S6: Overview of deletions supported by the long reads as compared to the reference *B. licheniformis* ATCC9789, with gene name and annotation of strain ATCC9789.

Gene	Annotation	Deletion (bp)	Coobra[1]	Stillspirits[1]	Browin[1]	Pureferm[1]	Strain[2]
CPQ91_RS01720	Serine protease	332	24	20	7	0	A
CPQ91_RS03295	polysaccharide deacetylase family protein (pdaC)	200	35	38	11	0	A
CPQ91_RS05620	S8 family peptidase	430	20	23	9	0	A
yqfD	Sporulation protein YqfD	728	22	21	11	0	A
sigF - spoIIAB	RNA polymerase sporulation sigma factor SigF - anti-sigma F factor SpoIIAB	151	67	68	89	100	B
amyS[3]	alpha-amylase	1,452 + 117[5]					
catA[3,4]	type A chloramphenicol O-acetyltransferase	746					

[1]When possible, the percentage of the long reads supporting the deletion is indicated for each sample, calculated as described in section 2.2.6.

[2]The data indicates the presence of two distinct sets of deletions, marked with either A or B, that are present at similar relative ratios in each sample.

[3]For *amyS* and *catA*, the coverage was much higher than the genome-wide average, and it was not possible to estimate a ratio at which the deletions were present

[4]deletion removed entire coding sequence of *catA*

[5]1,452 bp deletion, deleting almost the entire alpha-amylase encoding *amyS* gene and flanking this deletion, a second deletion of 117 bp downstream of *amyS*, and upstream of the adjacent gene predicted to encode a LacI family transcriptional regulator.

Table S7: Depth and breadth of coverage of short and long reads that map uniquely against reference genomes of the *Bacillus* species found in the metagenomic samples, as well as the two extrachromosomal elements; plasmid pFL7 of *B. licheniformis* and the putative prophage of *B. velezensis,* and the three transgenic constructs GMM protease1, pUB110-amylase, and GMM alpha-amylase2.

Reference			Coobra – normal depth	Coobra – high depth	Browin	Stillspirits	Pureferm
***B. licheniformis* ATCC9789**	**Short reads**	Depth	326	2,846	535	282	21
		Breadth	99	99	99	99	99
	**Long reads**	Depth	679		472	335	165
		Breadth	100		100	100	100
**construct GMM alpha-amylase2**	**Short reads**	Depth	58	421	141	61	37
		Breadth	21	81	30	39	20
	**Long reads**	Depth	252		136	144	176
		Breadth	100		94	100	100
***B. licheniformis* pFL7 plasmid**	**Short reads**	Depth	84	744	150	67	ND
		Breadth	100	100	100	100	ND
	**Long reads**	Depth	229		162	106	1
		Breadth	100		100	100	10
***B. amyloliquefaciens* DSM7**	**Short reads**	Depth	26	226	5	24	3
		Breadth	97	98	57	97	1
	**Long reads**	Depth		32	3	15	7
		Breadth		99	85	99	69
**construct GMM alpha-amylase1**	**Short reads**	Depth	851	2,983	205	829	945
		Breadth	44	51	38	46	40
	**Long reads**	Depth	1,336		80	695	2,023
		Breadth	100		100	100	100
***B. velezensis* 10075**	**Short reads**	Depth	3	21	2	3	170
		Breadth	26	96	6	26	97
	**Long reads**	Depth	4		1	2	1,088
		Breadth	94		35	82	98
***B. velezensis* GMM prophage**	**Short reads**	Depth	3	21	3	3	1,209
		Breadth	30	99	16	22	100
	**Long reads**	Depth	3		2	2	7,168
		Breadth	92		62	70	100
**construct GMM protease1**	**Short reads**	Depth	93	741	26	97	1,829
		Breadth	35	44	20	40	44
	**Long reads**	Depth	107		13	70	3,935
		Breadth	100		71	100	100

The depth of coverage is probably significantly underestimated by these numbers, in particular of GMM alpha-amylase2 and both pUB110-derived constructs since none of their sequence is unique in the sample, causing many reads derived from the constructs not to map uniquely. More specifically, the pUB110 sequence is present in both GMM protease1 and GMM alpha-amylase1, while the protease encoding insert from GMM protease1, the amylase encoding insert of GMM alpha-amylase1, and the complete GMM alpha-amylase2 are derived from the chromosomes of *B. velezensis*, *B. amyloliquefaciens*, and *B. licheniformis*, respectively.

ND: not detected

Table S8: Metrics of metagenomic long-read assemblies generated with Canu, and derived metagenomics assembled genomes (MAGs), obtained with Metabat2.

Metagenome or MAG	Total length (bp)	# contigs	Longest contig (bp)	Contig N50 (bp)	GC%	Completeness (%)	Taxonomic classification (GTDB-Tk)
Coobra - metagenome	9,108,307	143	669,224	312,463	46.38		
MAG 1	248,817	4	129,308	129,308	45.2	14.66	*B. amyloliquefaciens*
MAG 2	4,228,441	15	669,224	515,733	46.2	90.91	*B. licheniformis*
MAG 3	3,451,436	25	335,769	191,296	46.1	47.41	*B. amyloliquefaciens*
Stillspirits - metagenome	8,386,660	280	730,988	91,893	46.27		
MAG 1	4,088,567	10	730,988	511,124	46.2	72.41	*B. licheniformis*
MAG 2	3,235,791	165	113,929	27,849	46.5	64.66	*B. amyloliquefaciens*
Browin - metagenome	4,374,987	78	765,958	228,709	46.27		
MAG 1	4,208,849	25	765,958	228,709	46.1	91.32	*B. licheniformis*
Pureferm - metagenome	9,066,629	128	2,035,527	436,288	46.27		
MAG 1	4,238,192	25	742,550	235,309	46.1	91.05	*B. licheniformis*
MAG 2	3,950,764	6	2,035,527	2,035,527	46.1	73.68	*B. velezensis*

Table S9: Metrics of hybrid metagenomic assemblies from the mock metagenomic data sets (Supplementary text 6).

	short-read coverage	long-read coverage	Total length (bp)	# contigs	Longest contig (bp)	Contig N50 (bp)	GC%	Completeness (%)	Taxonomic classification
***10/1 B. amyloliquefaciens/B. velezensis* metagenome**			5,892,561	738	1,069,629	569,039	45.9	NA	NA
OPERA-MS MAGs/contigs									
MAG 1	263	53	3,649,602	6	1,069,629	794,111	46.6	80.7	*B. amyloliquefaciens*
contig displaying putative prophage *B. velezensis*	27	8	14,030	1	NA	NA	NA	NA	NA
Metabat2 MAGs									
MAG1*			1,892,274	3	794,111	604,747	46.5	57.89	*B. velezensis*
MAG2*			1,757,328	3	1,069,629	1,069,629	46.8	12.50	*B. velezensis*
MAG3*			221,778	2	181,869	181,869	44.1	0.00	*B. amyloliquefaciens*[1]
**10/1 *B. velezensis* /*amyloliquefaciens* metagenome**			8,282,922	1,612	1,284,947	772,874	46.3	NA	NA
OPERA-MS MAGs/contigs									
MAG1	133	937	4,876,916	11	1,284,947	1,230,058	46.0	98.2	*B. velezensis*
contig displaying putative prophage *B. velezensis*	289	1,867	13,873	1	NA	NA	NA	NA	NA
Metabat2 MAGs									
MAG1			3,234,314	3	1,284,946	1,176,493	46.1	65.52	*B. velezensis*
MAG2			1,572,778	7	489,773	345,927	46.3	31.58	*B. velezensis*
MAG3			472,265	2	437,079	437,079	45.9	4.17	*B. velezensis*[1]
MAG4			1,230,058	1	1,230,058	1,230,058	46.4	4.17	*B. velezensis*[1]
MAG5			391,450	3	153,783	119,487	47.9	19.3	*B. velezensis*

[1]GTDB-Tk did not assign a taxonomic label to this MAG (because it was too fragmented). Blastn was used to get an indication of the taxonomic classification

For the 10/1 *B. amyloliquefaciens/velezensis* assembly, the short-read dataset was composed of 250x *B. amyloliquefaciens* and 25x *B. velezensis* reads, and the long-read dataset consisted of 50x *B. amyloliquefaciens* and 5x *B. velezensis* reads. For the 10/1 *B. velezensis/amyloliquefaciens* assembly, the short-read dataset was composed of 220x *B. velezensis* and 22x *B. velezensis* reads, and the long-read dataset consisted of 1000x *B. velezensis* and 100x *B. amyloliquefaciens* reads (2.10). For each metagenome, the MAGs directly outputted by OPERA-MS by a reference-based clustering (i.e. supervised) approach are shown, together with the average short-read and long-read coverage that was obtained for each MAG. MAGs obtained by an alternative unsupervised binning tool, Metabat2, are presented as well. Taxonomic classification was done with GTDB-Tk. Additionally, metrics for contigs displaying the putative *B. velezensis* prophage are presented. NA = not applicable.

3. Supplementary text providing more detailed information concerning the bioinformatics analysis leading to the results and conclusions presented in the main text

Text S1. Characterization of viable strains isolated from samples Coobra and Pureferm

Microbial isolation experiments were performed to characterize any viable *Bacillus* strains contaminating the samples. This yielded isolates for samples Coobra and Pureferm, while from samples Stillspirits and Browin no viable strains could be retrieved. The obtained isolates were screened by qPCR to select the ones belonging to the *B. subtilis* group but not containing the protease construct, to exclude the *B. velezensis* GMM protease1 strain that was already characterized previously [8]. From the remaining isolates, 3 were chosen at random for short-read WGS for the Pureferm sample, while for the Coobra sample 10 isolates were retained for sequencing, to capture potential genetic variation. The main assembly statistics for these isolates are shown in Table S 4.

All three Pureferm isolate assemblies were classified as *B. velezensis* with GTDB-Tk. Additionally, their SNP addresses (Table S1) had at least the first 4 digits in common with each other, as well as with the previously characterized GMM protease1 isolates [8], indicating that the Pureferm isolates of the current study and the previously described GMM protease1 isolates are clonal strains. However, no AMR genes or pUB110 sequence were detected in the assemblies nor in the short read data of the new Pureferm isolates. This indicates that the three isolates represented the GMM protease1 host, but had lost the plasmid with the GMM construct due to the absence of antibiotic selective pressure during this isolation experiment.

The 10 Coobra isolates on the other hand were all classified with GTDB-Tk as *Bacillus licheniformis*. The SNP typing analysis produced identical SNP addresses

for the 10 isolates (Table S1), supporting that they were genetically identical and represented a single *B. licheniformis* strain. The Coobra isolate assemblies did not display contigs pointing to the presence of pUB110-derived plasmids. Additionally, no other conspicuous features were found that could indicate that this strain is genetically modified. Consequently, these results indicate that the Coobra *B. licheniformis* isolates may be natural strains, which may have end up in the samples either as a contamination of a natural producer organism, or as an accidental natural contamination. Alternatively, they may have carried a transgenic construct that was lost due to the absence of a suitable selection pressure. It is noteworthy that in a previous study [8] viable isolates of GMM protease1 were obtained from the Coobra sample, but in that case specific antibiotic selection pressure was applied to target strains carrying the pUB110-derived constructs, while in the current study the aim was a more open approach to investigate the presence of any culturable *Bacillus* strains.

Text S2. The GMM alpha-amylase1 construct is likely carried on an episomal high-copy plasmid

We could not establish with full certainty whether the GMM alpha-amylase1 construct was integrated into its *Bacillus* host genome, or if it was harbored as a free plasmid. Attempts to specifically isolate plasmid DNA from the FE matrix were unsuccessful, possibly due to considerable degradation of the DNA (results not shown), which renders plasmid-specific DNA extraction methods inefficient. Nevertheless, our observations strongly support that the GMM alpha-amylase1 construct exists as a free plasmid. None of the metagenomic assemblies displayed the construct as part of the chromosomal scaffold. Additionally, analysis of the long-read data of the samples did not reveal any reads with the transgenic construct flanked by *Bacillus* chromosomal sequences (results not shown). Nevertheless, transient chromosomal integration of the construct, such as was observed for GMM protease1

[7], is potentially also occurring for this construct. Lastly, the OPERA-MS assembly pipeline indicated that the depth of coverage for the transgenic construct was at least one order of magnitude higher than that observed for the *Bacillus* strains present in the samples, also supporting that the GMM alpha-amylase1 construct exists as a high-copy plasmid. For instance, for Coobra OPERA-MS reported a short- and long-read coverage for the GMM alpha-amylase1 construct of 39,804x and 2,558x, compared to a short- and long-read coverage of 292-293x and 46-48x, respectively, for the *B. amyloliquefaciens* MAGs. If the transgenic construct was integrated into the genome, these numbers would imply an integrated copy number of ~100 copies per genome, which is in theory feasible, but is reported to be highly unstable [1]. Moreover, literature reports that, in general, pUB110(-derived) plasmids replicate stably in *Bacillus* strains [57], and specifically also pKTH10 is reported to replicate as a free high-copy plasmid in *B. subtilis* [47].

Text S3. The Bacillus *contaminations constitute a considerable AMR gene load in the FE samples*

Table S4 and Table S5 provide an overview of the AMR genes and their depth of coverage detected in the metagenomic short-read and long-read data, respectively, which both show the same trends.

The genes *ant(4′)-la* and *bleO*, which are both harbored by the pUB110-derived transgenic constructs, were detected in all the samples at a high depth of coverage. Likewise, *catA*, the selection marker of the *GMM alpha-amylase2* construct, was abundantly present in all samples.

A class A beta-lactamase encoding gene *blaP*, encoding resistance to penicillins and specific to *B. licheniformis*, was found in all the samples. The *blaP* genes were a 100 % identical full-length match to the reference, indicating that they are complete and potentially functional. Investigation of the metagenomic assemblies showed that it was always associated with the *B. licheniformis* MAGs. Additionally, *blaP* was also present in

the *B. licheniformis* isolate assemblies, while it is also present in e.g. *B. licheniformis* strain ATCC9789, within an identical genomic context, confirming that it is of natural origin.

ermD, encoding erythromycin resistance, was also detected in the reads of Coobra, Stillspirits, and Browin. This gene is described as a fairly common trait of *B. (para)licheniformis* strains [50]. The detected *ermD* genes were a 100 % identical full-length match to the reference, indicating that they are complete and potentially functional. Contigs carrying *ermD* were assembled for the Coobra and Browin samples, for which web-based blastn resulted in almost 100% identical full-length matches with *B. licheniformis* genomes. Since *ermD* was not detected in the *B. licheniformis* isolate assemblies, we conclude that it is likely carried by one of the unculturable *B. licheniformis* strains.

Another AMR gene, *clbA*, was detected in both long- and short-read data of Coobra, and Stillspirits, as well as in the long-read data of Browin, albeit at a very low depth. *ClbA* is indigenous to *Bacillales* [58], and encodes 23S rRNA (adenine(2503)-C(8))-methyltransferase, conferring resistance to lincosamide, macrolide, and streptogramin antibiotics. However, the detected *clbA* genes showed only 93.05 % sequence identity to the reference, and displayed two single basepair deletions compared to the reference, causing frameshifts which render it doubtful that this was a functional AMR gene.

Finally, a few copies of *aph(3')-Ia* were found in the large long-read dataset of the Pureferm sample (Table S5). This *E. coli* gene encodes aminoglycoside O-phosphotransferase, conferring kanamycin resistance. A small fraction (~3,000 reads, 0.02 %) of the long reads from Pureferm were indeed classified as *E. coli* (results not shown). However, since the read abundance of this gene was very low and it was only detected in the error-prone long-read data set, it was not possible to verify whether this gene is likely to be intact and functional, thus presenting a potential risk.

Text S4. The B. licheniformis MAG in the alpha-amylase samples Coobra, Stillspirits and Browin represents two closely related B. licheniformis strains with deletions affecting their sporulation ability

Manual visual inspection of long-read alignments to the *B. licheniformis* ATCC9789 reference indicated that samples Coobra, Stillspirits, and Browin are contaminated with two closely related but distinct unculturable *B. licheniformis* strains. This is supported by the presence of two distinct sets of structural variants (SVs) in coding regions, which are all deletions that are absent in the reference strain, but are each supported by at least part of the long reads (Table S6, Figure S3, Figure S4, Figure S5). For example, in all samples, a significant number of long reads supported the presence of a deletion affecting the *sigF* and adjacent *spoIIAB* gene, but depending on the sample, the coverage at the site of this deletion ranged from 0 to 33% of the coverage of the flanking regions, while the remaining long reads indicated that there is no deletion at this site (Figure S4). These observations indicated the presence of (at least) two different unculturable *B. licheniformis* strains in samples Coobra, Stillspirits and Browin, one with a deletion in *sigF-spoIIAB*, and one where this deletion was not present. In sample Pureferm, the coverage at the site of the *sigF-spoIIAB* deletion was 0, supporting the presence of only a single strain. Likewise, evidence for four other deletions was found, each of them present in varying proportions in the different samples, in line with the presence of two *B. licheniformis* strains, designated A and B, in samples Coobra, Stillspirits, and Browin, and one single strain, i.e. B, in sample Pureferm (Table S6). Additionally, two more deletions were detected, in *catA* and *amyS* (Table S6). However, the coverage at these locations was much higher than the genome-wide average (further discussed in supplementary text 5). Therefore, it was not possible to estimate a ratio at which the deletions were present, and it was not evident which of the *B. licheniformis* strain carried these deletions, or whether it was present in both.

Web-based blastn analysis confirmed that none of the discovered deletions are present in a publicly available *B. licheniformis* genome, suggesting that they are genetic modifications.

To provide additional support for the deletions being really present, PCR was carried out on metagenomic DNA of samples Coobra and Pureferm, targeting the deletions in the genes implicated in spore formation for both strains, i.e. *yqfD in* strain A, and *sigF-spoIIAB in* strain B. This confirmed the presence of both the *yqfD* and *sigF-spoIIAB* deletions in sample Coobra, while in sample Pureferm only the latter deletion was detected (Figure S6, Figure S7).

In the *B. licheniformis* MAGs of the samples produced by the hybrid assembly, these deletions were not always apparent. In some samples, the genomic region containing a deletion was assembled but did not show any sign of a deletion (e.g. Figure S5), while in other samples the assembly simply failed, producing a contig breakpoint. Considering that the MAG in fact represents two different but likely closely related strains, this is not surprising, and it highlights that under these conditions the metagenomic assembly does not produce reliable results.

Text S5. Discovery and characterization of novel construct GMM alpha-amylase2

The GMM alpha-amylase2 construct was discovered after observing that for all samples, the long-read coverage of the coding region of the alpha-amylase encoding gene *amyS* of *B. licheniformis* and its immediate up- and downstream regions was at least 4-fold higher than the average coverage of the reference *B. licheniformis* ATCC 9789 genome (Figure S8). Furthermore, most read alignments did not extend beyond this region, but showed supplementary alignments at an entirely different location in the reference, i.e. at the site of *catA*, where the read coverage was also much higher than average, similar to that of *amyS* (Figure S9). Since *amyS* and *catA* are not naturally flanking genes, this suggests an unnatural association and hence a hitherto

uncharacterized unauthorized GMM contamination. However, no contigs representing this construct were present in any of the hybrid metagenomic assemblies. As an alternative, metagenomic assemblies were made using only the long reads (Table S8), which produced contigs displaying the complete GMM alpha-amylase2 construct for all four samples. More specifically, the long-read assembly of Pureferm displayed a number of contigs that indicated that the *GMM alpha-amylase2* construct was integrated into a *B. licheniformis* host genome at the site of the wild-type *catA* gene. Based on the sequence of the Pureferm contigs, PCR assays targeting all the unnatural associations of the construct were carried out for the Coobra and Pureferm samples (Figure S 10), confirming that the contigs correctly represented the GMM alpha-amylase2 construct and its genomic location (Figure 2).

We could not determine the exact copy number at which the construct has been integrated into the host genome, although it encompasses at the very least two copies based on the raw long reads and long-read assemblies. Based on the ratio of the long-read abundance of the construct compared to the average depth of coverage of the *B. licheniformis* host reference, the copy number of the integrated GMM alpha-amylase2 construct would likely be at least 3. We cannot exclude the possibility that the construct was also integrated at other locations in the genome despite finding no such apparent other associations in the data. Concerning the host strain, since Pureferm only contains *B. licheniformis* strain B, this strain almost certainly carries the GMM alpha-amylase2 construct. However, since there is no sample available that is only contaminated with strain A and not strain B, we could not establish whether or not *B. licheniformis* strain A harbors the GMM alpha-amylase2 construct.

Text S6. The B. velezensis GMM protease1 genome likely collapses with that of the unculturable B. amyloliquefaciens (GMM alpha-amylase1) strain in all the assemblies

We have demonstrated previously the presence of viable *B. velezensis* GMM protease1 strains in the samples Coobra and Pureferm [8]. However, the presence of this GMM was not clearly confirmed in the metagenomic analysis of the Coobra sample, since only the very high-depth dataset MetaBAT2 produced two low-quality MAGs classified as *B. velezensis* (Table 3). Only in the metagenomic assembly of Pureferm, which is labeled as a protease FE product and showed the highest contamination level according to the qPCR signal for this GMM, a high-quality MAG classified as *B. velezensis* was detected. However, as Table S3 shows, in all the metagenomic assemblies contigs were detected that support the presence of the GMM protease1 construct. Additionally, in all the assemblies contigs were present that matched at least a significant fraction of a putative extrachromosomal prophage that is associated with the *B. velezensis* GMM, which was confirmed through read mapping analysis (Table S7). Furthermore, read mapping analysis of the metagenomic samples also provided support for the presence of the *B. velezensis* GMM genome, albeit at a very low read abundance (Table S7). Together, these observations indicate that *B. velezensis* GMM contaminations are present in all four samples, also in samples Browin and Stillspirits, from which a viable *B. velezensis* GMM strain was not isolated. Likewise, although the Pureferm assembly did not contain a MAG for the *B. amyloliquefaciens* GMM alpha-amylase1, read mapping analysis indicated that this strain is also represented in the Pureferm sample, albeit at a low read abundance (Table S7).

Since the GMM protease1 could be isolated as a viable strain from the Coobra and Pureferm samples, it was unexpected that the relative read abundance of this strain was so low in the Coobra sample, and that we could not obtain a good-quality MAG for the *B. velezensis* GMM even in the Coobra sample, despite the very high short-read depth at

which this sample was sequenced. *Bacillus* strains typically survive as spores, and even a few remaining spores can result in the isolation of a viable strain from the sample. Together with the fact that our DNA extraction was not tailored specifically to extract DNA from the thick-walled *Bacillus* spores, the low read abundance is perhaps not that surprising.

To explain the lack of a MAG for the *B. velezensis* GMM in the Coobra, Stillspirits and Browin assemblies, and for a *B. amyloliquefaciens* MAG in the Pureferm assembly, we considered that *B. velezensis* and *B. amyloliquefaciens* are closely related sister species [59]. Their ANI, the average nucleotide identity of all orthologous genes shared between genomes of both species, is close to 94%, while the cutoff commonly used for ANI-based species delineation is 95% [22]. When two closely related species such as *B. velezensis* and *B. amyloliquefaciens* are present, especially when their read abundance is highly uneven (Table S7), the reads of the underrepresented strain/species can be unrecognized by the assembler as representing a distinct genome. Those reads are then 'collapsed' into the MAG of the more highly abundant strain/species, resulting in the underrepresented strain being incompletely assembled through fragmented contigs representing regions where the sequence similarity is relatively low [60,61].

To assess whether this might explain our observations, we carried out hybrid metagenomic assemblies on mock samples, composed of reads from a publicly available dataset from a *B. amyloliquefaciens* isolate, and reads from the *B. velezensis* GMM isolate available from our previous study [8]. In the first mock dataset, the reads were mixed at a ~10/1 *amyloliquefaciens/velezensis* ratio and a total read-depth similar to that suspected of the Coobra sample, in order to mimic the conditions of the Coobra metagenomic hybrid assembly. This confirmed that under these conditions, the assembly pipeline could not properly separate both genomes, as OPERA-MS outputted only one MAG, classified as *B. amyloliquefaciens*, at a calculated read-depth similar to that for the input *B. amyloliquefaciens*

and *B. velezensis* short-read data combined (Table S9). Nevertheless, a contig was obtained displaying an almost full-length 100% identical match to the putative prophage identified in the *B. velezensis* isolates, similar to what we found in the Coobra and Stillspirits samples (Table S3).

In the second mock dataset, the situation was reversed, to approximate the conditions prevailing in the Pureferm dataset, i.e. a 10/1 ratio of *B. velezensis* and *B. amyloliquefaciens* reads, and an absolute read depth scaled to that of the Pureferm dataset. Again, the OPERA-MS pipeline outputted only one MAG, classified as *B. velezensis*, indicating that the presence of a *B. amyloliquefaciens* strain would remain unnoticed under these conditions.

Similar to the GMM protease1, the question arises why the viable *B. licheniformis* strain was not assembled as a separate MAG in the metagenomic hybrid assembly for the Coobra sample. We hypothesize that the presence of three *B. licheniformis* strains in the sample, with the unculturable ones being present at a much higher read abundance than the isolate, which is possibly only present as a few spores, presumably lead to an assembly collapse into one MAG for *B. licheniformis*, hiding the presence of the isolable *B. licheniformis* strain in the metagenomic assembly.

Text S7. Challenges and bottlenecks of the bioinformatics analysis

Although the use of a combination of short- and long-read sequencing and a hybrid assembly strategy was of clear added value, providing valuable insights in the microbial composition of the samples, several additional and hands-on analyses were still required to resolve the composition of the samples. The average length of the long reads obtained from the metagenomic DNA extract was quite low, presumably due to degradation of the DNA in the samples, limiting to some extent the advantage of using long-read data. Nevertheless, the long reads were of pivotal importance to prove that two distinct

unculturable *B. licheniformis* strains were present in the samples, and to detect and characterize the novel GMM alpha-amylase2 construct.

One main challenge was caused by the presence of closely related strains in the samples, especially because some of the strains were only represented at a very low read abundances. This caused a (partial) collapse of the assemblies of the closely related *B. amyloliquefaciens* and *B. velezensis* genomes, and of the unculturable and culturable *B. licheniformis* strains. While strain-level resolution of metagenomic data is currently still a bottleneck for most established assembly tools, novel developments in the field of bioinformatics indicate that this can be overcome with dedicated tools, provided sufficiently high-depth metagenomic sequencing data is available [61,62].

Additionally, detection and characterization of the GMM alpha-amylase2 transgenic construct was not straightforward. The OPERA-MS pipeline starts with an initial short-read assembly, after which long reads are employed to improve the assembly result, and resulted in assemblies lacking this construct. In this complex case study, it turned out to be of added value to additionally perform assemblies based on long reads only. The long-read assembler Canu, despite the resulting assemblies generally being inferior to those of the hybrid assembler OPERA-MS, did succeed in producing contigs with the GMM alpha-amylase2 construct for all samples.

Additional work will be required to develop a suitable pipeline allowing to investigate the relationship and potential contamination source of the GMM contaminations based on metagenomic data. For more in-depth typing and source tracking of GMM contaminants, tools for strain resolution based on read mapping to a reference database could be considered [62]. However, reference-based tools require the availability of a suitable reference database, which is not straightforward in case of unauthorized GMM, in which case *a priori* information is generally scarce. Alternatively, tools that are tailored to strain-aware *de novo* assembly could be applied, although these

generally require very deep sequencing to reliably detect strains present at low abundance [62].
